# Safety, Immunogenicity and Dose Ranging of a New Vi-CRM_197_ Conjugate Vaccine against Typhoid Fever: Randomized Clinical Testing in Healthy Adults

**DOI:** 10.1371/journal.pone.0025398

**Published:** 2011-09-30

**Authors:** Pierre van Damme, Froukje Kafeja, Alessandra Anemona, Venere Basile, Anne Katrin Hilbert, Ilse De Coster, Simona Rondini, Francesca Micoli, Rana M. Qasim Khan, Elisa Marchetti, Vito Di Cioccio, Allan Saul, Laura B. Martin, Audino Podda

**Affiliations:** 1 Center for the Evaluation of Vaccination, Vaccine & Infectious Disease Institute, University of Antwerp, Antwerp, Belgium; 2 Novartis Vaccines Institute for Global Health, Siena, Italy; 3 Novartis Vaccines & Diagnostics, Clinical Serology Laboratories, Marburg, Germany; Health Protection Agency, United Kingdom

## Abstract

**Background:**

Typhoid fever causes more than 21 million cases of disease and 200,000 deaths yearly worldwide, with more than 90% of the disease burden being reported from Asia. Epidemiological data show high disease incidence in young children and suggest that immunization programs should target children below two years of age: this is not possible with available vaccines. The Novartis Vaccines Institute for Global Health developed a conjugate vaccine (Vi-CRM_197_) for infant vaccination concomitantly with EPI vaccines, either starting at 6 weeks with DTP or at 9 months with measles vaccine. We report the results from a Phase 1 and a Phase 2 dose ranging trial with Vi-CRM_197_ in European adults.

**Methodology:**

Following randomized blinded comparison of single vaccination with either Vi-CRM_197_ or licensed polysaccharide vaccines (both containing 25·0 µg of Vi antigen), a randomised observer blinded dose ranging trial was performed in the same center to compare three concentrations of Vi-CRM_197_ (1·25 µg, 5·0 µg and 12·5 µg of Vi antigen) with the polysaccharide vaccine.

**Principal Findings:**

All vaccines were well tolerated. Compared to the polysaccharide vaccine, Vi-CRM_197_ induced a higher incidence of mild to moderate short lasting local pain. All Vi-CRM_197_ formulations induced higher Vi antibody levels compared to licensed control, with clear dose response relationship.

**Conclusions:**

Vi-CRM_197_ did not elicit safety concerns, was highly immunogenic and is therefore suitable for further clinical testing in endemic populations of South Asia.

**Trial Registration:**

ClinicalTrials.gov NCT01123941 NCT01193907

## Introduction

Typhoid fever, a disease caused by *Salmonella enterica* serovar Typhi (*S*. Typhi), represents a global public health issue causing over 21 million cases of disease and over 200,000 deaths per year worldwide [1]. Although endemic in many developing countries, typhoid fever has the highest incidence in the Indian subcontinent where 90% of the *S*. Typhi related morbidity and mortality are reported [1], [2]. Typhoid fever was previously considered a typical disease of school children and adolescents [3]; however, most recent epidemiologic data from India, Pakistan, Bangladesh and Vietnam clearly show that younger children are also highly affected by typhoid fever and are at risk of severe disease [3]–[7]. These data have prompted a re-consideration of prevention strategies and suggest that immunization of subjects below two years could be a better approach to protect young children, to increase vaccination coverage and to reduce costs by concomitant delivery with routine vaccines of the Expanded Program on Immunization (EPI) [8]–[10].

Currently available typhoid vaccines, although moderately effective and well tolerated, cannot be administered to children younger than two years. Additionally, the Vi polysaccharide induces a short lasting protection and requires repeated boosters which might cause hypo-responsiveness [11]. Since *S.* Typhi is an obligate human pathogen, an effective vaccination program could lead to substantial herd protection [12]. Therefore, new highly efficacious vaccines are needed to have the greatest public health impact in high risk areas and to allow their use in existing EPI vaccination programs. As shown by clinical results with the Vi-*r*EPA vaccine in Vietnam [13]–[15], a conjugate Vi vaccine appears to meet these criteria and, in addition, clinical data generated in Vietnam have allowed the establishment of a serological threshold for clinical protection [13].

The Novartis Vaccines Institute for Global Health (NVGH) was founded in 2007 with the not for profit mission of developing effective and affordable vaccines for neglected infectious diseases of impoverished communities of developing countries [16]. By conjugation of the Vi polysaccharide with the CRM_197_ protein, NVGH developed a vaccine against typhoid fever (Vi-CRM_197_) that has the potential to induce a T-cell dependent immunological memory and to be effectively used for infant immunization in developing countries [17]–[19].

We describe results from a Phase 1 trial and a subsequent Phase 2 dose ranging trial aimed to evaluate the clinical profile of Vi-CRM_197_ in European adults and to select the dose for further clinical testing in endemic countries, where the vaccine will be ultimately used.

## Materials and Methods

### Study vaccine

Vi-CRM_197_ contained purified Vi polysaccharide, derived from *Citrobacter*, chemically conjugated to mutant diphtheria toxin carrier protein, CRM_197_, widely used as carrier protein for several conjugate vaccines [17]–[19].

The vaccine clinical lot (VI-10-001), available in single dose vials, contained 25 µg of Vi antigen conjugated to CRM_197_ in 0.5 mL of injectable solution and did not contain any adjuvant, stabilizer or preservative.

### Study design

Phase 1 and 2 studies were designed as randomised controlled trials and were conducted sequentially at the Center for the Evaluation of Vaccination of the University of Antwerp (Antwerp, Belgium) from May to November 2010.

Healthy adults aged 18 to 40 years were enrolled in both studies. Subjects were excluded from the trial if they presented with serious chronic or progressive disease, with immune dysfunction, under immunosuppressive therapy, with bleeding diathesis, with hypersensitivity to vaccine components, with progressive or severe neurological disorder, with seizures or Guillain-Barré syndrome, with malignancy or lymphoproliferative disorder, that previously received vaccines against typhoid fever, with previous history of infection with *S.* Typhi, that received blood products, with body temperature ≥38.0°C within 3 days of study vaccination. Women who were pregnant or breast-feeding and women of childbearing age not willing to use acceptable birth control measures were also excluded. Ethical approval was obtained from the Ethical Committee of the University Hospital of Antwerp. The studies were conducted according with the International Conference on Harmonization, Good Clinical Practice guidelines (ICH-GCP). All participants provided written informed consent.

In Phase 1, Vi-CRM_197_ vaccine was administered as single 0.5 mL dose containing 25 µg of Vi antigen. Subsequent to the Phase 1 study, with the objective to define the optimal Vi-CRM_197_ concentration for further development, the Phase 2 dose ranging trial was implemented, using three different concentrations administered as single 0.5 mL doses containing 1.25 µg, 5.0 µg and 12.5 µg of Vi respectively; the formulations were obtained by bedside dilution of the 25 µg vaccine with appropriate amount of saline solution. In both studies the control vaccine was a licensed Vi polysaccharide (Vi-PS) vaccine (Typherix®, GlaxoSmithKline) containing 25 µg of Vi.

In the Phase 1 study, 50 subjects were randomized, with a 1∶1 ratio using a randomization block size of four, to receive either Vi-CRM_197_ 25 µg or Vi-PS as a single intramuscular (IM) injection in the deltoid of the non-dominant arm. In the Phase 2 study, 88 subjects were randomized, with a 1∶1∶1∶1 ratio using randomization block size of four, to receive a single IM dose of one of three Vi-CRM_197_ concentrations (1.25 µg, 5.0 µg and 12.5 µg of Vi respectively) or the Vi-PS. In both studies, randomization sequence was computer generated by the sponsor. Assignment of subject to treatment group was performed by designated unblinded clinical site personnel by means of individual envelopes provided by the sponsor who also established ad hoc monitoring visits during both trials to ensure appropriateness of all protocol procedures. The protocols for the two trials and supporting CONSORT checklist are available as supporting information; See Checklist S1, Protocol S1 and Protocol S2.

### Evaluation of immunogenicity

Blood samples of 20 mL were obtained for hematological and hematochemical analyses (Phase 1 only) prior to vaccination (day 1) and 28 days post vaccination. For both studies, urine samples were obtained prior to vaccination to evaluate potential drug abuse; additionally, urine pregnancy tests were performed in females before vaccination and at the study end. Blood samples of 10 mL were obtained from both Phase 1 and Phase 2 subjects on days 1, 28, and on day 180 in Phase 1 only. Blood samples were centrifuged within 24 hours and serum was maintained below −20°C.

Antibody levels were measured by an enzyme-linked immunossorbent assay (ELISA) in the Novartis Vaccines & Diagnostics human serology laboratory (Marburg, Germany), using Vi polysaccharide from *Citrobacter*. Round bottom 96-well microtiter plates were coated overnight with 1.0 µg/mL Vi in PBS pH 7.0, plates were blocked with 5% skim milk powder in PBS, then duplicate samples of human sera, serially diluted 2-fold, were added and incubated for 2 hours at room temperature. Goat anti-human IgG (γ-chain specific) conjugated to alkaline phosphatase was added and incubated 2 hours at room temperature. After 30 min incubation with p-nitrophenyl-phosphate solution, the reaction was stopped. The absorbance at 405 nm and 650 nm was read. The A_405_–A_650_ were used as the test parameter.

Each plate contained a serially diluted pool of anti-Vi human sera. This pool was assigned a value of 115.4 ELISA U/mL. A standard curve was generated from the corresponding values using a 4 parameter fit. ELISA units in test sera were calculated from this curve. Low and high human serum controls were included on each plate. The ELISA lower detection limit ranged from 3.0 to 7.8 U/mL.

### Evaluation of safety

Safety of study vaccines was assessed by evaluation of solicited indicators of reactogenicity within 7 days after vaccination. Following vaccination, subjects were observed at the site for at least 30 minutes. Subjects maintained a daily diary card for 7 days post-vaccination recording local reactions (pain, induration, erythema), systemic reactions (headache, malaise, chills, myalgia, arthralgia, fatigue), body temperature (fever defined as axillary temperature ≥38.0°C) and use of analgesic and antipyretic drugs. Local pain and systemic reactions were scored by severity (mild, moderate and severe) and erythema and induration by the maximum diameter per day. All adverse events (AEs) were recorded for 28 days post-vaccination, while all serious adverse events (SAEs) were assessed and recorded for the entire study duration. For consistent measurement of reactions, subjects were provided with digital thermometers for measurement of body temperature and with a plastic ruler for measurement of size of local reactions.

### Statistical methods

No statistical null hypothesis was associated with immunogenicity or safety objectives. In the Phase 1 study with 25 subjects per group, there was a 90% probability of observing at least 1 subject with an adverse event if the true rate of such an event was 8.7%. In the Phase 2 study with 20 subjects per vaccine group, there was a 90% probability of observing at least 1 subject with an adverse event if the true rate of such an event was 10.9%.

All analyses were descriptive in nature. The pre-specified primary population for immunogenicity analysis was the Modified Intention to Treat Population (MITT). The MITT Population included all subjects who received the vaccination, had at least one post-vaccination blood sample collected, and at least one ELISA result available.

Seroconversion was defined as a post vaccination increase of at least four times the pre-vaccination antibody concentration.

Geometric mean concentrations (GMCs) and associated two-sided 95% confidence intervals (CIs) were calculated by exponentiating (base 10) the means of log-transformed (base 10) titers and their 95% confidence intervals. Antibody concentrations below assay limit of detection were assigned a value of one half of the cut-off. At each sampling time GMCs of antibodies (with two-sided 95% CIs) and reverse cumulative curves were calculated. The percentages of subjects with seroconversion were calculated for each vaccine group in each study. Analyses were performed using SAS version 9.1 (SAS Institute Inc, Cary, North Carolina).

## Results

**Figure 1:**
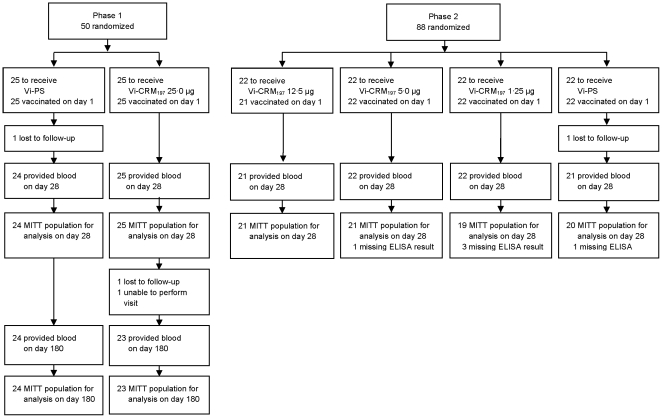
Subject Completion Flow Chart.

A total of 138 subjects were enrolled across both studies (50 Phase 1; 88 Phase 2), of whom 133 (96%) completed the studies [47 (94%) Phase 1; 86 (98%) Phase 2]. There were no withdrawals from either study due to AEs (figure 1). Demographic characteristics were similar among vaccine groups and between the two studies (table 1). None of the subjects showed clinically significant variations of haematological and hematochemical parameters after vaccination (day 28) compared to pre-vaccination values. The most frequently reported local reaction was mild to moderate local pain, which occurred with a similar frequency among all four Vi-CRM_197_ groups and was consistently higher in Vi-CRM_197_ groups compared with Vi-PS control groups. Percentages of subjects reporting pain did not show a dose dependent relation in the Vi-CRM_197_ groups. One subject in each of the four Vi-CRM_197_ groups reported severe pain lasting <1 day; no severe cases of erythema and induration were reported (table 2). Percentages of subjects reporting systemic reactions were similar among all vaccine groups. Headache and fatigue were the most commonly reported systemic reactions in all vaccine groups (overall range was 27% to 48% and 24% to 50% for headache and fatigue respectively). Severe systemic reactions were uncommon and were reported by seven subjects overall (one case in the Vi-CRM_197_ 25 µg group and six in Vi-PS group) (table 1). No differences were observed in the use of analgesic or antipyretic drugs among study groups (table 2). Of all study subjects, 59% of the Vi-CRM_197_ and 53% of the Vi-PS recipients reported at least one AE during the 28 days after vaccination and 38% and 26% of these were considered possibly or probably related to the study vaccine, in the Vi-CRM_197_ and Vi-PS groups respectively. In Phase 1, no possibly or probably related AEs were reported from day 29 to day 180. Most of the AEs reported in both studies were mild or moderate in nature. The percentage of subjects reporting any AEs or AEs possibly or probably vaccine related was lower in Phase 1 than in Phase 2 (table 3). Two unrelated SAEs were reported in Phase 1 study in the Vi-PS group, one hospitalization for pneumothorax and one hospitalization for ankle fracture. No SAEs were reported in the Phase 2 study. No AEs leading to premature withdrawal from the study and no deaths were reported in both studies.

**Table 1:** Baseline demographic characteristics by vaccine group.

	Phase 1			Phase 2				
	Vi-PS	Vi-CRM_197_ 25 µg	All	Vi-CRM_197_ 12.5 µg	Vi-CRM_197_ 5.0 µg	Vi-CRM_197_ 1.25 µg	Vi-PS	All
	n = 25	n = 25	n = 50	n = 22	n = 22	n = 22	n = 22	n = 88
Mean age (SD)	23.9 (4.6)	21.9 (2)	22.9 (3.7)	24.3 (4.9)	24.3 (5.4)	24 (5.7)	23.8 (5.5)	24.1 (5.3)
Number of women (%)	15 (60)	18 (72)	33 (66)	14 (64)	15 (68)	17 (77)	15 (68)	61 (69)
Ethnic origin, no. (%)								
Caucasian	25 (100)	25 (100)	50 (100)	22 (100)	22 (100)	22 (100)	21 (95)	87 (99)
Black	0	0	0	0	0	0	1 (5)	1 (1)
Mean weight (SD)	72.5 (13.4)	71.9 (12.3)	72.2 (12.7)	70 (14.2)	70.1 (12.1)	69.2 (10.5)	70.7 (8.6)	70 (11.3)
Mean height (SD)	174.8 (8.3)	173.6 (9)	174.2 (8.6)	173.1 (9.6)	173.0 (9.9)	172.2 (7.3)	175.5 (7.0)	173.5 (8.5)
BMI	23.7 (4)	23.8 (2.9)	23.7 (3.4)	23.3 (3.9)	23.4 (3.5)	23.3 (3)	22.9 (2)	23.2 (3.2)

Note: BMI, Body Mass Index; SD, standard deviation.

**Table 2:** Number (%) of Subjects Reporting Local and Systemic Reactions During Days 1 to 7.

		Phase 1		Phase 2			
		Vi-PS	Vi-CRM_197_ 25 µg	Vi-CRM_197_ 12.5 µg	Vi-CRM_197_ 5.0 µg	Vi-CRM_197_ 1.25 µg	Vi-PS
		n = 25	n = 25	n = 21	n = 22	n = 22	n = 22
Local reactions, no. (%)	Severity						
Erythema	Any	1 (4)	4 (16)	3 (14)	2 (9)	2 (9)	0
	>100 mm	0	0	0	0	0	0
Induration	Any	1 (4)	7 (28)	3 (14)	4 (18)	3 (14)	2 (9)
	>100 mm	0	0	0	0	0	0
Pain	Any	9 (36)	21 (84)	17 (81)	16 (73)	20 (91)	8 (36)
	Severe*	0	1 (4)	1 (5)	1 (5)	1 (5)	0
Systemic reactions, no. (%)							
Chills	Any	0	1 (4)	1 (5)	2 (9)	2 (9)	2 (9)
	Severe*	0	0	0	0	0	0
Malaise	Any	4 (16)	9 (36)	4 (19)	4 (18)	4 (18)	4 (18)
	Severe*	2 (8)	0	0	0	0	1 (5)
Myalgia	Any	0	7 (28)	4 (19)	3 (14)	3 (14)	3 (14)
	Severe*	0	0	0	0	0	0
Arthralgia	Any	0	1 (4)	2 (10)	0	0	0
	Severe*	0	0	0	0	0	0
Headache	Any	8 (32)	9 (36)	10 (48)	9 (41)	9 (41)	6 (27)
	Severe*	1 (4)	1 (4)	0	0	0	0
Fatigue	Any	6 (24)	12 (48)	7 (33)	11 (50)	8 (36)	7 (32)
	Severe*	2 (8)	0	0	0	0	0
Fever ≥38°C	Any	0	0	0	0	0	0
	≥40°C	0	0	0	0	0	0
Analgesic/Antipyretic Medication Use		5 (20)	3 (12)	5 (25)	7 (27)	7 (32)	3 (14)

*Defined as unable to perform normal daily activity.

**Table 3:** Number (%) of Subjects with Adverse Events During Days 1 to 28.

	Phase 1		Phase 2			
	Vi-PS	Vi-CRM_197_ 25 µg	Vi-CRM_197_ 12.5 µg	Vi-CRM_197_ 5.0 µg	Vi-CRM_197_ 1.25 µg	Vi-PS
	n = 25	n = 25	n = 21	n = 22	n = 22	n = 22
Any AE	10 (40)	8 (32)	11 (52)	16 (73)	18 (82)	15 (68)
Vaccine-related AEs*	3 (12)	6 (24)	5 (24)	11 (50)	12 (55)	9 (41)
AEs leading to discontinuation	0 (0)	0 (0)	0 (0)	0 (0)	0 (0)	0 (0)
Any SAE	2 (8)	0 (0)	0 (0)	0 (0)	0 (0)	0 (0)
Vaccine-related SAEs*	0 (0)	0 (0)	0 (0)	0 (0)	0 (0)	0 (0)

Note:

*Determined by the investigator to be possibly or probably related to the study vaccine.

**Figure 2:**
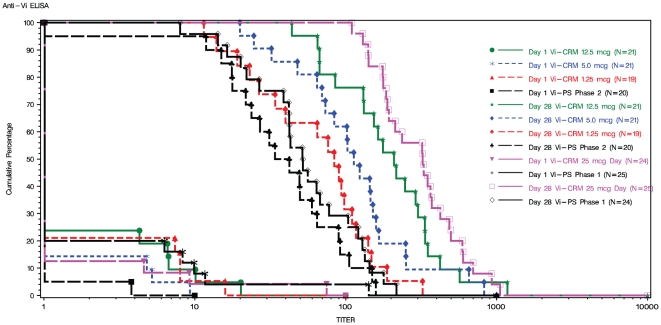
Reverse Cumulative Curves of Anti-Vi ELISA Concentrations before (day 1) and four weeks (day 28) After Vaccination with Vi-CRM_197_ or Vi-PS. Antibody concentrations are given in log scale. Values below the limit of detection of the assay were set to 1.0 U/mL.

A total of 130 subjects had evaluable day 28 anti-Vi ELISA results and were included in the immunogenicity analyses at day 28 post-vaccination. At baseline (day 1), a high percentage of the sera (84%) had antibody levels below limit of detection. These sera were assigned values half the detection level and thus the calculated GMCs, which were similar in all the six vaccine groups, reflect the assigned values (table 4). The GMCs at day 28 of the four Vi-CRM_197_ groups were higher (range from 63 to 304) than the licensed Vi-PS vaccine groups (range from 37 to 52) (Table 3). Day 28 GMCs in the Vi-CRM_197_ groups were dose dependent; the highest Vi-CRM_197_ dose (25 µg) elicited the highest antibody level (304) and the lowest level (63) was observed for the Vi-CRM_197_ 1.25 µg dose. These findings are further illustrated by reverse cumulative distribution curves for anti-Vi ELISA concentrations (figure 2). Four weeks after vaccination, at least 95% of the subjects vaccinated with Vi-CRM_197_ achieved seroconversion, whereas 88% and 95% of subjects achieved seroconversion in the Vi-PS control groups. In the Phase 1 study, six months after the vaccination (day 180), seroconversion rates were still very high in both vaccine groups (95% and 92% in the Vi-CRM_197_ 25 µg and Vi-PS groups, respectively). However, although the GMC of Vi-CRM_197_ 25 µg group (69) was still higher than that of Vi-PS (51), a faster decrease in antibody levels compared to the control group was noticed (table 4).

**Table 4:** Immunogenicity Profile of NVGH Vi-CRM_197_ Conjugate Vaccine and Licensed Vi-PS Vaccine.

		Phase 1		Phase 2			
		Vi-PS	Vi-CRM_197_ 25 µg	Vi-CRM_197_ 12.5 µg	Vi-CRM_197_ 5.0 µg	Vi-CRM_197_ 1.25 µg	Vi-PS
**Time point**	**GMCs**						
day 1	GMC	3.8	3.2	3.0	2.4	2.8	2.2
	95% CI	(2.7, 5.3)	(2.3, 4.6)	(2.4, 3.8)	(1.9, 3.0)	(2.2, 3.6)	(1.7, 2.8)
	N	25	24	21	21	19	20
day 28	GMC	52	304	192	111	63	37
	95% CI	(38, 71)	(226, 411)	(129, 286)	(75, 165)	(41, 95)	(24, 55)
	N	24	25	21	21	19	20
day 180	GMC	51	69	na	na	na	na
	95% CI	(37, 69)	(50, 94)				
	N	24	23				
	**Seroconversion** *						
day 28	n (%)	21 (88)	23 (96)	21 (100)	21 (100)	18 (95)	19 (95)
	95% CI	(68, 97)	(79, 100)	(84, 100)	(84, 100)	(74, 100)	(75, 100)
	N	24	24	21	21	19	20
day 180	n (%)	22 (92)	21 (95)	na	na	na	na
	95% CI	(73, 99)	(77, 100)				
	N	24	22				

*Number and percentage of subjects achieving at least a four-fold rise in ELISA antibody concentrations in the post-vaccination blood sample.

na: not applicable.

## Discussion

Epidemiological studies provide compelling evidence that typhoid fever induces significant disease burden in Asia and that, particularly in the urban slums of the Indian subcontinent, the disease incidence is huge [1]–[2]. Additionally, the most recent data do not support the previous belief that typhoid is a disease affecting almost exclusively school children and adolescents [3]; in fact, children aged less than two years are highly affected in India, Pakistan and Bangladesh by clinically severe disease [3]–[6]. In industrialized countries, typhoid control has been largely achieved by provision of clean water, adequate sanitation and overall improved hygiene conditions. Unfortunately these conditions are only a long term solution for most of the developing countries where the disease is endemic [6], [8]. This public health issue is further complicated by the increasing emergence of multi-drug resistant strains of *S.*Typhi [6], [8]–[9]. Resistance to antimicrobials renders the control of the disease even more difficult and expensive, as the easily available and cheaper first-line antibiotics are not effective and their use leads to increased hospitalization and associated costs [6], [8], [10]. Therefore, vaccine prevention represents the only concrete option for efficient disease control.

After discontinuation of the highly reactogenic whole-cell *S.* Typhi vaccine [6], effective and well tolerated vaccines, such as Vi polysaccharide and live attenuated Ty21a vaccine, have been developed. However, these vaccines have limitations; the Vi polysaccharide vaccine cannot be administered to subjects younger than 2 years and, even in pre-school children, its efficacy is controversial [10]. Additionally, polysaccharide vaccines do not induce immunological memory, require frequent boosters, to maintain adequate protection, and may be associated with hyporesponsiveness [11]. The commercially available formulation of Ty21a vaccine can only be administered to children older than 6 years and requires three doses to induce protection. Consequently the use of these vaccines and their impact on the control of the disease in endemic countries has generally been very limited. The development of new vaccines has been advocated to overcome the limitations of the current ones and allow the use of alternative vaccination strategies for younger children and infants [6], [8]–[9]. Although the National Institutes of Health (NIH) pioneering work has shown that a prototype Vi conjugate vaccine can be developed and is highly efficacious, there has not been a sufficient commercial incentive for vaccine manufacturers to invest in the development of improved typhoid vaccines for developing countries.

NVGH, an institute created with the specific objective of developing new and affordable vaccines against neglected diseases for the impoverished populations of developing countries [16], has developed a Vi conjugate vaccine which, in preclinical studies, has shown to be safe and highly immunogenic [17]–[18].

The clinical studies described in this paper have shown that Vi-CRM_197_ is clinically well tolerated. The only evidence of reactogenicity was given by the higher incidence of pain at the injection site compared to Vi-PS. Interestingly, the reaction rates did not show a dose dependent relationship and were similar across the four Vi-CRM_197_ concentrations tested. However, in most of the cases, reported pain was mild to moderate and of short duration. Additionally, pain was never associated with any evidence of inflammation at the injection site, as shown by the very low rates of erythema and induration, which were not different between the two study vaccines. The use of analgesic medications in the conjugate vaccine group was low and similar to that of the Vi-PS control group; finally, no subject withdrew from the trial due to adverse reactions. Systemic post immunization reactions were consistent with an injectable vaccine; their rates were generally low and did not significantly differ among the various vaccine groups. Importantly, no case of fever was reported, nor was any case of vaccine related serious adverse event. Larger Phase 2–3 studies will help define more precisely the safety profile of the vaccine, but the initial data show that Vi-CRM_197_ is a safe vaccine.

The immunogenicity data show that the Vi-CRM_197_, even at very low antigen concentrations (i.e., 1.25 µg/dose = 1/20^th^ of the licensed control concentration), is similar or better than Vi-PS (25 µg/dose) and the overall postimmunization Vi-CRM_197_ to Vi-PS GMC ratio varies between 1.7 and 5.8. As shown by field trials with the Vi-PS [20]–[21] and with the Vi-*r*EPA conjugate vaccine [13], antibodies to the Vi antigen confer protection against typhoid fever. Therefore, inferring the efficacy of the conjugate vaccine based on immunogenicity data is scientifically justified and demonstration that antibody levels induced by Vi-CRM_197_ in infants are not less than those induced by the Vi-PS vaccine in older children, a population where vaccine efficacy has been shown, could be an appropriate basis for regulatory approval. However, establishing a correlation with the immunogenicity of the Vi-*r*EPA vaccine and, more importantly, with the serological threshold of protection defined in the Vi-*r*EPA Vietnamese efficacy trial [13] would be extremely valuable and is currently underway.

The current data are very promising and suggest that an effective conjugate vaccine against typhoid fever can be rapidly developed. This vaccine has been primarily developed for immunization of the impoverished populations of South Asia, which have the highest risk of infection and severe disease. Therefore, it is essential to evaluate its clinical profile in the populations that will ultimately use the vaccine. To this purpose, age de-escalation Phase 2 clinical trials with Vi-CRM_197_ are now ongoing in South Asia.

## Supporting Information

Protocol S1 Trial Protocol of Phase 1 Trial
(PDF)

Protocol S2 Trial Protocol of Phase 2 Trial
(PDF)

Checklist S1 CONSORT Checklist
(DOC)
