# Supplementary material for: Safety, Immunogenicity and Dose Ranging of a New Vi-CRM197 Conjugate Vaccine against Typhoid Fever: Randomized Clinical Testing in Healthy Adults
Source: PLoS One. 2011 Sep 30;6(9):e25398. doi: 10.1371/journal.pone.0025398 (PMC3184126; doi:10.1371/journal.pone.0025398)
Supplement: Protocol S1 — Trial Protocol of Phase 1 Trial (PDF) [file pone.0025398.s001.pdf]

CLINICAL STUDY PROTOCOL H01_01TP

Version#1 of 25 November, 2009

Amendment#1 of 14 December 2009, Version#2

Amendment#2 of 27 January 2010, Version#3

Amendment#3 of 26 April 2010, Version#4

Amendment#4 of 15 September 2010, Version#5

EudraCT No 2009-017453-35

A Phase I, Randomized, Single–blind, Controlled, Single Center Study to Evaluate the Safety and Immunogenicity of the NVGH Glycoconjugate Vaccine against S. Typhi in Adult Subjects 18 to 40 Years of Age.

Property of Novartis Vaccines Institute for Global Health

Confidential

May not be used, divulged, published or otherwise disclosed without written consent of Novartis Vaccines Institute for Global Health.

STUDY PERIOD: Each subject will participate into the study for 180 days after vaccination.

RATIONALE:

Despite the availability of S.Typhi vaccines for older children and adults, typhoid fever remains a major health problem in developing countries. The World Health Organization (WHO) estimated the global typhoid disease burden at 21 million cases annually with 200 to 600 thousand deaths per year[1]. In many areas of Asia, Africa and Latin America, high incidence rates for S.Typhi have been reported and this disease remains a major public health problem. Of those infected, children of school age or younger are disproportionately affected. Serious complications occur in up to 10% of the cases, resulting in death in 1% to 4% of younger children.

The currently licensed vaccines against S.Typhi are the oral live attenuated and the Vi polysaccharide vaccines. As purified Vi polysaccharide is a T independent antigen, the

currently licensed vaccines are not able to prime vaccinees for immunological memory, can not be boosted by repeated vaccination[2] and are poorly immunogenic in the very young children; therefore they are only recommended for children $\geq$2 years of age . The coupling of polysaccharide antigens to carrier proteins transforms the antigen, so called conjugate polysaccharide, into a T-cell dependent antigen, capable of inducing an immunological memory and an adequate immune response in infants and young children[3].

NVGH Vi-CRM_{197} is a glycoconjugated vaccine, based on chemical conjugation of the Vi polysaccharide with the CRM_{197} carrier protein. Due to the T-cell-dependent immunological properties of glycoconjugates, this vaccine should be able to overcome the limitations of the currently available polysaccharide vaccines and offer an effective tool for immunization of not only young children but also infants and toddlers less than 2 years of age, a longer-lasting immune response and a boostable memory response against S. Typhi. Consequently the NVGH Vi-CRM conjugate vaccine may be integrated into EPI routine infant vaccination.

The NVGH candidate Vi-CRM_{197} vaccine results from the conjugation of two well characterized components: the Vi polysaccharide (which is a widely licensed vaccine) and the mutant of the diphtheria toxin protein (CRM_{197}), a carrier protein used in many licensed vaccines, including the *H. influenzae* type b, *N. meningitidis* and *S. pneumoniae* vaccines. The NVGH Vi antigen is obtained from a strain of *Citrobacter* -----------, unlike the licensed Vi polysaccharide vaccine which is derived from a Salmonella Typhi strain. The Vi molecules obtained from the two strains are chemically undistinguishable.

The proposed Phase I trial is therefore aimed to evaluate the safety and immunogenicity profiles of a new Vi-CRM_{197} conjugate vaccine in healthy human adults in comparison with the currently licensed Vi polysaccharide vaccine (obtained from a S. Typhi strain).

OBJECTIVES:

Safety Objectives

To evaluate the safety profile of investigational NVGH Vi-CRM_{197} conjugate vaccine against that of the licensed Vi polysaccharide vaccine by measuring rates of post immunization reactions and adverse events, and incidence of serious adverse events.

Immunogenicity Objectives

To evaluate the immunogenicity profile of investigational NVGH Vi-CRM_{197} conjugate vaccine against that of the licensed Vi polysaccharide vaccine as measured by enzyme-linked immunosorbent assay (ELISA), at 28 days and 6 months post-immunization.

METHODOLOGY:

This will be a randomized, single blind, active vaccine-controlled, single center, Phase 1 clinical trial.

During the screening period (day -7 to day -1), subjects giving informed consent will be screened for general health status by obtaining a 20 mL blood draw for hematological and hematochemical tests. Urinalysis tests will also be performed. For all women the urinalysis test will include the evaluation of human chorionic gonadotropin (hCG) to exclude pregnancy.

No pharmacokinetic tests will be performed as evaluation of pharmacokinetic properties is not required for vaccines unless new delivery systems are employed or when the vaccine contains novel adjuvants or excipients [EMEA/CHMP/VWP/164653/2005].

Only subjects with screening tests within normal values and women with negative pregnancy test will be randomized. An additional blood draw of 20 mL for hematological and hematochemical tests will be obtained and urinalysis will be repeated at 28 days after vaccination.

Enrolled subjects will be randomized at a 1:1 ratio to receive, at day 1 a single intramuscular injection of either the investigational NVGH Vi-CRM_{197} conjugate vaccine or the licensed Vi polysaccharide vaccine, using a syringe with a 25 mm needle. Each randomized subject will have 10 mL of blood drawn for serology at day 1 following randomization, and will be vaccinated with the assigned vaccine after the blood draw. Another 10 mL of blood will be drawn for serology at day 28 post immunization and at 6 months after immunization (see Appendix I).

After immunogenicity data at 28 days become available, for the purpose of creating a standard reference serum and standardizing the serological assay, selected volunteers with high antibody titers from the two study groups will be asked to provide a blood sample of up to 100 ml. At the start of the study (Visit 1) the additional voluntary blood donation will be discussed with the subject, explaining the aims and the benefit/risk.

Reactogenicity will be assessed daily during the first week following vaccination: local and systemic reactions occurring at day 1 to day 7 will be collected and recorded in the diary card. The overall safety follow-up will be 6 months (180 days) for each subject: all adverse events (AE) will be collected and documented for 28 days; serious adverse events (SAE) will be collected and documented for the duration of the study (180 days).

Summary of Study Vaccination and Blood Sampling

Groups	***Vaccine***	***Number of subjects***	***Day 1***	***Day 28***	***Day 180***
Group A	*NVGH Vi-CRM_{197}*	*25*	*Blood sampling*	*Blood sampling*	*Blood sampling*

	conjugate vaccine		*Vaccination*		
Group B	*Licensed Vi polysaccharide vaccine*	*25*	*Blood sampling Vaccination*	*Blood sampling*	*Blood sampling*

NUMBER OF SUBJECTS PLANNED

A total of 63 subjects are planned for enrollment into the study to have 50 vaccinated (25 for each of the two study groups). Subjects withdrawn or lost to follow up will not be replaced.

SUBJECTS POPULATION

The study population will consist of male and female adult subjects aged 18 to 40 years. Female subjects must use birth control during screening period and study participation. Those with immunosuppressive conditions including the use of chronic high dose inhaled steroids will not be included.

INCLUSION CRITERIA

1. Males and females of age ≥18 to ≤40 years.
2. Individuals who, after the nature of the study has been explained to them, have given written consent according to local regulatory requirements.
3. Individuals in good health as determined by the outcome of medical history, physical examination, hematological / hematochemical blood tests and urinalysis and clinical judgment of the investigator.
4. If women, a negative pregnancy test and willingness to use birth control measures for the entire study duration.

EXCLUSION CRITERIA

1. Individuals with behavioral or cognitive impairment or psychiatric disease that, in the opinion of the investigator, may interfere with the subject's ability to participate in the study.
2. Individuals with any progressive or severe neurological disorder, seizure disorder or Guillain-Barré syndrome.
3. Individuals who are not able to understand and to follow all required study procedures for the whole period of the study.
4. Individuals with history of any illness that, in the opinion of the investigator, might interfere with the results of the study or pose additional risk to the subjects due to participation in the study.

5. Individuals with known or suspected HIV infection or HIV related disease, with history of an autoimmune disorder or any other known or suspected impairment /alteration of the immune system, or under immunosuppressive therapy including use of systemic corticosteroids or chronic use of inhaled high-potency corticosteroids within the previous 30 days, or were in chemotherapy treatment within the past 6 months.
6. Individuals with a known bleeding diathesis, or any condition that may be associated with a prolonged bleeding time.
7. Individuals with any serious chronic or progressive disease according to judgment of the investigator (e.g., neoplasm, insulin dependent diabetes, cardiac, renal or hepatic disease).
8. Individuals who have any malignancy or lymphoproliferative disorder.
9. Individuals with history of allergy to vaccine components.
10. Individuals participating in any clinical trial with another investigational product 30 days prior to first study visit or intent to participate in another clinical study at any time during the conduct of this study.
11. Individuals who have previously received any vaccines against typhoid fever (either oral live attenuated or injectable vaccines)
12. Individuals who received any other vaccines within 4 weeks prior to enrollment in this study or who are planning to receive any vaccine within 4 weeks from the study vaccine
13. Individuals who have received blood, blood products and/or plasma derivatives including parenteral immunoglobulin preparations in the past 12 weeks.
14. Individuals who are part of study personnel or close family members to the personnel conducting this study.
15. Individuals with body temperature $\geq$ 38.0 degrees Celsius within 3 days of intended study immunization.
16. BMI > 35 $kg/m^{2.}$
17. Individuals with history of substance or alcohol abuse within the past 2 years.
18. Women who are pregnant or breast-feeding or of childbearing age who have not used or do not plan to use acceptable birth control measures, for the duration of the study.
19. Females with history of stillbirth, neonatal loss, or previous infant with anomaly.
20. Individuals who have a previously ascertained or suspected disease caused by S. Typhi.
21. Individuals who have had household contact with/and or intimate exposure to an individual with laboratory confirmed S. Typhi.

22. Any condition which, in the opinion of the investigator may interfere with the evaluation of the study objectives.

VACCINES

<u>Test Vaccine</u>

The test vaccine is NVGH S. Typhi glyconjugate vaccine.

A 0.5 mL dose of Vi-CRM vaccine contains 25 µg of Vi; 25 µg of CRM_{197} and 29 mM of Sodium phosphate buffer.

One 0.5 mL dose out of a single dose vial of NVGH Vi-CRM vaccine will be injected IM in the deltoid of the non-dominant arm.

<u>Reference Vaccine</u>

Reference vaccine is the Vi polysaccharide vaccine (Typherix®,GlaxoSmithKline). A 0.5 mL dose of Typherix® contains 25 µg of Vi.

One 0.5 mL dose out of single dose pre-filled syringes of Vi vaccine will be injected IM in the deltoid of the non-dominant arm.

<u>Concomitant Vaccines or Treatment</u>

None.

SEROLOGY

The immunogenicity of the vaccines will be assessed by ELISA assay. The serologic assays on clinical samples will be performed at Novartis Vaccines, Clinical Serology Laboratory, Marburg, Germany or a delegated laboratory.

CRITERIA FOR EVALUATION

Immunogenicity Endpoints

The measures of immunogenicity, against the Vi antigen of S. Typhi, will include:

- Geometric mean concentrations (GMCs), pre- and post-vaccination, as determined by ELISA, and applicable geometric mean ratios between post- and pre-vaccination samples.

- Seroconversion rate: percentage of subjects achieving at least a four-fold rise in ELISA antibody titer in the post-vaccination blood sample

- Seroprotection rate: percentage of subjects achieving an ELISA titer ≥ 7 units, the estimated threshold of protection[4] in the post-vaccination blood sample.

Safety Endpoints (criteria for assessing safety endpoints)

The measures of safety will include:

- Deviations from normal values (see Appendix II) of hematological and haematochemical blood tests and urinalysis after immunization. Clinical significance of abnormal values will be assessed by medical judgment.

- Numbers and percentage of subjects with solicited local and systemic adverse reactions as well as numbers and percentage of subjects with reported unsolicited adverse events and serious adverse events.

Solicited local reactions include erythema, induration and pain at injection site; solicited systemic reactions include headache, arthralgia, chills, fatigue, malaise, myalgia, and fever as measured by axillary temperature for Day 1 through 7 of the study.

All local and systemic reactions will be collected for 7 days after immunization (i.e., days 1 to 7). All AEs will be collected for 28 days and all SAEs will be collected throughout the entire study (180 days).

STATISTICAL METHODS:

Statistical Hypothesis

This Phase I safety and immunogenicity trial is aimed to descriptively evaluate the safety and immunogenicity profiles of the study vaccines. No specific hypotheses are tested in this trial.

Sample Size and Power Considerations

Given the sample size selected for this trial, the precision to define certain incidence rates and the power to detect differences in the incidence rates between the vaccine groups in ad hoc or exploratory analyses will be indicated in the following examples:

- For each vaccine, with 25 subjects per vaccine group, there is a 90% probability of observing at least 1 subject with an adverse event if the true rate of such an event is 8.7%.

  The probability to detect at least one subject with an adverse event (for each of the vaccine groups) is reported in Table 1 by underlying rates of AEs descending from 15% - 1.5%:

Table 1

**Underlying Event Rate (Frequency of AE)**	**Probability of Detection N=25**
15%	98%
12.5%	96%
10%	93%
7.5%	86%
5%	72%
2.5%	47%
1.5%	31%

- Comparative statistics for the safety variables will not be calculated, the study has low power to detect anything other than large differences in the incidence of local and systemic reactions between vaccination groups. In fact, a difference between a 10% reaction rate (n=25) in one of the vaccine groups and a 20% reaction rate in one of the other vaccine groups (n=25) is detectable with only 8% power at a 5% two-sided alpha level. Safety comparisons, with an 80% power and a 5% two-sided alpha level, will permit to detect differences (calculated by using the Fisher exact test) as reported in Table 2:

Table 2

**Underlying Event Rate in one vaccine group**	**Difference detected with a 80% probability**
30%	41.0%
20%	40.7%
10%	38.5%
5%	34.0%
2.5%	31.5%

- The precision of the estimates, expressed by the 95% Clopper-Pearson CI, for several possible observed values of the percentages of seroprotection and seroconversion for all vaccine groups is shown in Table 3:

Table 3

Response Rate<br>%	95% CI<br>(N = 25)<br>% %
48	27.8 - 68.7
52	31.3 - 72.2
56	34.9 - 75.6
60	38.7 - 78.9
64	42.5 - 82.0
68	46.5 - 85.1
72	50.6 - 87.9
76	54.9 - 90.6
80	59.3 - 93.2
84	63.9 - 95.5
88	68.8 - 97.5
92	74.0 - 99.0
96	79.6 - 99.9
100	86.3 - 100.0

- In performing an immunogenicity comparison, a sample sizes of 25 per group will permit to detect a 2.5-fold difference in antibody titer with 93% power at alpha=0.05 when the standard deviation of $\log_{10}$ titers is no greater than 0.4.

INTERIM ANALYSIS

Preliminary analyses are planned after Day 28 data become available to evaluate safety and immunogenicity at 4 weeks after immunization in order to better plan the subsequent trials of the clinical development plan. Individual subject results from unblinded preliminary analyses will not be made available to site personnel until the end of the study.

DATA SAFETY MONITORING BOARD

A data safety monitoring board (DSMB) will not be utilized for this study.

APPENDIX I - TIMES AND EVENTS TABLE

**Study Periods**	**Screening**	**Vaccination**	**Post-Vaccination**	**Blood donation**	**Follow-up**
**Visit No.**	**1**	**2**	**3**	**4**	**5**
**Clinic Visit? (Yes/No)**[a]	Yes	Yes	Yes	Yes	Yes
**Study Day**	-7 to -1	1	28	70	180
**Study Visit Window**	NA	NA	+7	+15	+15/-15
ICF	x				
Exclusion/Inclusion	x	x[b]			
Medical history	x	x[b]			
Physical examination[c]	x	x[b]	x		x
Randomization		x[b]			
**Vaccine administered**		x			
Serology Blood draw [max 10 mL]		x[b]	x		x
Urinalysis	x		x		
Pregnancy test	x	x			x
Safety Laboratory Blood draw [max 20 mL]	x		x		
Blood draw for standard reference serum [max 100 mL]				x	
Diary Card Dispensed		x			
Diary Card Collected and/or Reviewed			x		
Assess Local/ Systemic Reactions[d]		x			
Assess AEs and SAEs [e]		x	x		x
Concomitant medications[f]	x	x	x		x
Ter Study mination[g]					x

a. Clinic visit “no” refers to telephone contact only with subject
b. Performed prior to vaccination
c. Physical examination (including injection site or intended injection site) must be performed by a qualified health professional designated within the Site Responsibility Delegation Log. Complete physical examination will be performed at Screening, Day 1, Day 28 and Day 180
d. Data on local and systemic reactions will be collected by the study personnel for all subjects at 2 hours post-injection. Subjects will record local and systemic reactions on the diary card daily for 7 days after study vaccination
e. Assess AEs and SAEs according to Safety Assessment Table (Table 6.2.1)
f. Collect concomitant medications/vaccines according to Safety Assessment Table (Table 6.2.1)
g. Any subject who terminates the study earlier is recommended to undergo study-termination procedures.

APPENDIX II - HEMATOLOGICAL, HAEMATOCHEMICAL BLOOD TESTS AND URINALYSIS TABLE - ACCEPTED RANGE OF LABORATORY PARAMETERS

**HEMATOLOGY**		
Erythro Sedimentation Rate 1st h	F 0-20<br>M 0-15	mm/h
White Blood Cells	F/M 3.7 – 10.0	10^3/µl
Red Blood Cells	F 3.80 – 5.20<br>M 4.10 – 5.30	milj/µl
Haemoglobin	F 11.6-15.6<br>M 12.9-17.2	g/dl
Haematocrit	F 34 – 46<br>M 38 – 50	%
Platelets	F/M 15-37	10^3/µl
Neutrophils segmented	F/M 45-75	%
Eosinophils	F/M < 5	%
Basophils	F/M < 2	%
Monocytes	F/M 1-10	%
Lymphocytes	F/M 18-45	%
Prothrombin time (Quick)	F/M 70 – 100	%
**CLINICAL CHEMISTRY**		
Total Cholesterol	F/M <190	mg/dl
Triglycerides	F/M <180	mg/dl
Total bilirubin	F/M <1.1	mg/dl
Aspartic Aminotransferase (ASAT/GOT)	F <31<br>M <37	U/l
Alanine Aminotransferase (ALAT/GPT)	F <31<br>M <41	U/l
γ-Glutamyl Transpeptidase (γ-GT)	F <32<br>M<49	U/l
Lactic Dehydrogenase (LDH)	F/M <480	U/l
Alkaline Phosphatase (AP)	F <104<br>M <129	U/l
Total Proteins	F/M 6.6-8.7	g/dl
Glucose random, not fasting	F/M 100-126	mg/dl
Urea	F/M <50	mg/dl
Creatinine	F <0.90<br>M <1.20	mg/dl
Sodium	F/M 135 – 145	mEq/l

Potassium	F/M 3.5 – 5.1	mEq/l

**URINE ANALYSIS**	
Human chorionic gonadotropin (hCG)	Negative
Glucose	Negative or Trace
Proteins	Negative or Trace
Bilirubin	Negative
Urobilinogen	Negative
pH	4,5-8,0
Specific Gravity	1015-1032
Haemoglobin	Negative or Trace
Ketones	Negative
Nitrites	Negative
**MICROSCOPIC TEST on urine**	
Leucocytes	<25/µl
Erythrocytes	<25/µl
Round Epithelial Cells	Negative or Trace
Casts	Negative
Crystals	Negative
Mucus	Negative or Trace
Bacteria	Negative or Trace
**VIROLOGY**	
HbsAg	Negative
Hepatitis C antibodies	Negative
HIV antibodies	Negative
**DRUGS OF ABUSE in urine**	
Opiate	Negative (cut-off 300 ng/ml)
Cocaine	Negative (cut-off 300 ng/ml)
Amph/Metamphetamine	Negative (cut-off 1000 ng/ml)
Cannabinoides	Negative (cut-off 50 ng/ml)

TABLE OF CONTENTS

RATIONALE: 1
OBJECTIVES: 2
METHODOLOGY: 3
NUMBER OF SUBJECTS PLANNED 4
SUBJECTS POPULATION 4
VACCINES 6
SEROLOGY 6
CRITERIA FOR EVALUATION 6
STATISTICAL METHODS: 7
APPENDIX I - TIMES AND EVENTS TABLE 10
APPENDIX II - HEMATOLOGICAL, HAEMATOCHEMICAL BLOOD TESTS AND URINALYSIS TABLE - ACCEPTED RANGE OF LABORATORY PARAMETERS 11
LIST OF ABBREVIATIONS AND DEFINITIONS OF TERMS 16
1.0 BACKGROUND AND RATIONALE 17
2.0 OBJECTIVES 18
2.1 Safety Objectives 18
2.2 Immunogenicity Objectives 18
3.0 STUDY DESIGN AND INVESTIGATIONAL PLAN 18
3.1 Overview of Study Design 18
3.2 Discussion of Overall Study Design 19
3.3 Study sites 20
3.4 Target Population 20
3.5 Recruitment 20
3.3 Study Procedures and Flowchart 21
3.3.4 Vaccine Supply, Storage, Tracking and Labeling 26
3.3.5 Processing, Labeling and Storage of Serum Samples for Serology 27
3.4 Duration of Subject’s Expected Participation in the Entire Study 28
3.5 Stopping/Pausing Rules 28
4.0 SELECTION OF STUDY POPULATION 28
4.1 Inclusion Criteria 28

4.2 Exclusion Criteria 28
4.3 Withdrawal of Subjects from Therapy or Assessment 30
5.0 TREATMENT OF SUBJECTS 30
5.1 Investigational Vaccine - NVGH S. Typhi conjugate vaccine 31
5.2 Control Vaccine – GSK Typherix® 31
5.3 Concomitant Vaccines or Treatment 31
5.4 Vaccines Preparation and Administration 31
5.5 Other Concomitant Treatment or Vaccines 32
5.6 Vaccination Compliance 33
6.0 IMMUNOGENICITY AND SAFETY ASSESSMENTS 33
6.1 Appropriateness of Measurements 33
6.1.1 Immunogenicity 33
6.1.2 Methods, Criteria and Timing for Assessing and Recording Efficacy/Immunogenicity Parameters 34
6.2 Safety Parameters 34
6.2.1 Local and Systemic Reactions 35
6.2.2 Adverse Events 35
6.2.3 Serious Adverse Events 37
6.2.4 Subject’s Diary Card 38
6.2.6 Methods and Timing for Assessing and Recording Safety Parameters 38
6.4 Data Safety Monitoring Board 40
7.0 STATISTICAL PLAN 40
7.1 Statistical Hypothesis 40
7.2 Sample Size and Power Considerations 40
7.3 Population for Analysis 42
7.4 Analysis of Demographic and Baseline Characteristics 43
7.5 Analysis of Efficacy/Immunogenicity Endpoints 43
7.5.1 Description of Response Variables 43
7.5.2 Statistical Methods for Efficacy/Immunogenicity Variables 43
7.6 Analysis of Safety (Endpoints) and Tolerability 44
7.6.1 Analysis of Extent of Exposure 44

7.6.2 Analysis of Local and Systemic Reactions 44
7.6.3 Analysis of Other Adverse Events 45
7.7 Planned Interim Analysis 45
8.0 STUDY MONITORING, AUDITING AND DOCUMENTATION 45
8.1 Study Monitoring 46
8.2 Source Data Verification 46
9.0 DATA MANAGEMENT 46
9.1 Data Handling Procedures 47
9.2 Documentation of Study Findings 47
9.3 Data Protection 48
10.0 RECORD RETENTION 48
11.0 USE OF INFORMATION AND PUBLICATION 48
12.0 ETHICS 48
13.0 REFERENCE LIST 50

LIST OF ABBREVIATIONS AND DEFINITIONS OF TERMS

Ab	Antibody
AE	Adverse Event
ALAT/GPT	Alanine Aminotransferase
AP	Alkaline Phosphatase
ASAT/GOT	Aspartic Aminotransferase
BCDM	Biostatistics and Clinical Data Management
CRA	Clinical Research Associate
CRF	Case Report Form
CRM	Cross-Reacting Material
DCF	Data Clarification Form
DSMB	Data Safety Monitoring Board
EC	Ethics Committee
eDC	electronic Data Capture
ELISA	Enzyme-Linked Immunosorbent Assay
EPI	Expanded Program on Immunization
GCP	Good Clinical Practice
GMC	Geometric Mean Concentration
GMP	Good Manufacturing Practice
HEE	Hidden Entry Envelopes
ICF	Informed Consent Form
ICH	International Conference on Harmonization
IEC	Indipendent Ethics Committee
IM	Intramuscular
IND	Investigational New Drug
IRB	Institutional Review Board
LDH	Lactic Dehydrogenase
MD	Medical Doctor
MedDRA	Medical Dictionary for Regulatory Activities
mITT	Modified Intention-To-Treat
nAb	neutralizing Antibody
NVGH	Novartis Vaccines Institute for Global Health
PP	Per Protocol
REB	Regional Ethics Board
SAE	Serious Adverse Event
SOP	Standard Operating Procedure
SUSAR	Suspected Unexpected Serious Adverse Reaction
WHO	World Health Organization
γ-GT	γ-Glutamyl Transpeptidase

1.0 BACKGROUND AND RATIONALE

Salmonella is a rod-shaped gram-negative facultative anaerobe bacterium belonging to the *Enterobacteriaceae* family. *Salmonella enterica* serovar Typhi (S. Typhi) is pathogenic exclusively for humans and cause systemic infections and typhoid fever, a severe, contagious and lifethreatening systemic disease which may result in persistent fever with or without severe complications[5;6].

Despite the availability of S. Typhi vaccines for older children and adults, typhoid fever remains a major health problem in developing countries. The World Health Organization (WHO) estimated the global typhoid disease burden at 21 million cases annually with 200 to 600 thousand deaths per year[1]. In many areas of Asia, Africa and Latin America, high incidence rates for S.Typhi have been reported and this disease remains a major public health problem. Of those infected, children of school age or younger are disproportionately affected. Serious complications occur in up to 10% of the cases, resulting in death in 1% to 4% of younger children.

The existence of multidrug-resistant S. Typhi, that was found to be resistant to treatment with most of commonly used antibiotics such as chloramphenicol, ampicillin, streptomycin and tetracycline, is a serious and growing problem in the treatment of typhoid, especially in the developing country, where the vaccination has been proven to be an effective way of controlling typhoid, expecially in vulnerable group[5].

The currently licensed vaccines against S. Typhi are the oral live attenuated and the Vi polysaccharide vaccines. As purified Vi polysaccharide is a T independent antigen, the currently licensed vaccines are not able to prime vaccines for immunological memory, can not be boosted by repeated vaccinations[2] and are poorly immunogenic in the very young children; therefore they are only recommended for children $\geq$2 years of age . The coupling of polysaccharide antigens to carrier proteins transforms the antigen, so called conjugate polysaccharide, into a T-cell dependent antigen, capable of inducing an immunological memory and an adequate immune response in infants and young children[3].

NVGH Vi-CRM_{197} is a glycoconjugated vaccine, based on chemical conjugation of the Vi polysaccharide with the CRM_{197} carrier protein. Due to the T-cell-dependent immunological properties of glycoconjugates, this vaccine should be able to overcome the limitations of the currently available polysaccharide vaccines and offer an effective tool for immunization of not only young children but also infants and toddlers less than 2 years of age, a longer-lasting immune response and a boostable memory response against S. typhi. Consequently the NVGH Vi-CRM conjugate vaccine may be integrated into EPI routine infant vaccination.

The NVGH candidate Vi-CRM_{197} vaccine results from the conjugation of two well characterized components: the Vi polysaccharide (which is a widely licensed vaccine) and the mutant of the diphtheria toxin protein (CRM_{197}), a carrier protein used in many

licensed vaccines, including the *H. influenzae* type b, *N. meningitidis* and *S. pneumoniae* vaccines. The NVGH Vi antigen is obtained from a strain of *Citrobacter* -----------, unlike the licensed Vi polysaccharide vaccine which is derived from a *Salmonella* Typhi strain. The Vi molecules obtained from the two strains are chemically undistinguishable.

As this is a phase 1 study, no previous clinical data are available. A comprehensive review of NVGH Vi-CRM_{197} conjugate vaccine, including pre-clinical data, is contained in the Investigator's Brochure supplied by Novartis Vaccines Institute for Global Health; this document should be reviewed prior to initiating the study.

As for other vaccines, mutagenic or carcinogenic potential have not been evaluated for the NVGH Vi-CRM_{197} vaccine. The safety, tolerability and lack of toxicity demonstrated in preclinical studies were considered adequate to support the clinical testing of this vaccine in males and non pregnant females.

The proposed Phase I trial is aimed to evaluate the safety and immunogenicity profiles of a new Vi-CRM_{197} conjugate vaccine in healthy human adults in comparison with the currently licensed Vi polysaccharide vaccine (obtained from a S. Typhi strain).

The trial will be conducted in compliance with the protocol, GCP and the applicable regulatory requirements.

2.0 OBJECTIVES

2.1 Safety Objectives

To evaluate the safety profile of investigational NVGH Vi-CRM_{197} conjugate vaccine against that of the licensed Vi polysaccharide vaccine by measuring rates of post immunization reactions and adverse events, and incidence of serious adverse events.

2.2 Immunogenicity Objectives

To evaluate the immunogenicity profile of investigational NVGH Vi-CRM_{197} conjugate vaccine against that of the licensed Vi polysaccharide vaccine as measured by enzyme-linked immunosorbent assay (ELISA), at 28 days and 6 months post-immunization.

3.0 STUDY DESIGN AND INVESTIGATIONAL PLAN

3.1 Overview of Study Design

This is a phase I, randomized, single blind, controlled study in adult women and men evaluating the safety and immunogenicity of a NVGH glycoconjugate vaccine against S. Typhi.

The study has four periods: Screening, vaccination and two follow up periods.

- Screening period (Visit 1): Subjects, who provide a written informed consent, will be screened for eligibility at -7 to -1 day prior vaccination.

- Vaccination period (Visit 2): eligible subjects will be randomized to receive one of two vaccines. Subjects will receive one injection on Day 1.

- Short term follow up period (up to Visit 3): Subjects will be followed for 28 (+7) days after vaccination.

- Long term follow up period (up to Visit 5): Subjects will be followed for 180 (-15/+15) days after vaccination.

After immunogenicity data at 28 days become available, for the purpose of creating a standard reference serum and standardizing the S. Typhi serological assay, selected volunteers from the two study groups with high antibody titers will be asked to provide a blood sample of up to 100 mL. This additional voluntary blood donation will be discussed with the subject, explaining the aims and the benefits, at Visit 1.

In order to better plan the subsequent trials of the clinical development plan, preliminary analyses are planned after Day 28 data become available, to evaluate safety and immunogenicity at 4 weeks after immunization. Individual subject results from unblinded preliminary analyses will not be made available to site personnel until the end of the study.

3.2 Discussion of Overall Study Design

This phase I study utilizes several standard features of clinical study design intended to reduce bias, including the random assignment of subjects to treatment groups. Because the study vaccine and the reference vaccine have a different appearance, a single-blind design was chosen. The subjects will be blinded to the vaccine received.

The active comparator, the Vi polysaccharide vaccine, was selected to enable comparison of the study treatment to a similar licensed polysaccharide vaccine, since there is no other S.Typhi conjugate vaccine currently available. There are no placebo groups in this study since there would be no immunogenic response and no local or systemic reactions, which would enable a comparison with the properties of the Vi-CRM vaccine.

The frequency of study visits and assessments are consistent with previous studies investigating the tolerability and immunogenicity of conjugate vaccines. In those studies, the majority of local and systemic reactions occurred within 2 days of vaccination and lasted no more than 4 days and therefore any local or systemic reactions in this study should be captured during Visit 2 and Visit 3.

3.3 Study sites

The study will be performed at one study site:

Administrative part of the study will be performed at:
Centre for the Evaluation of Vaccination

The study will be performed at:
Research Unit of the University Hospital

Campus 3 Eiken Universiteitsplein, 1 (loc. R2.14), B-2610 Antwerpen
www.ua.ac.be/cev
www.vhpb.org

3.4 Target Population

The study will be performed in adults 18-40 years of age. A total of 63 subjects are planned for enrollment into the study to have 50 vaccinated.

3.5 Recruitment

The Centre for the Evaluation of Vaccination existing database of study volunteers will be used. Further recruitment will be performed through intranet system of the University of Antwerp and the university hospital of Antwerp.

Posters will be posted in buildings of the University of Antwerp as well as the University Hospital. Advertisements on local Antwerp TV channel will be also shown.

3.3 Study Procedures and Flowchart

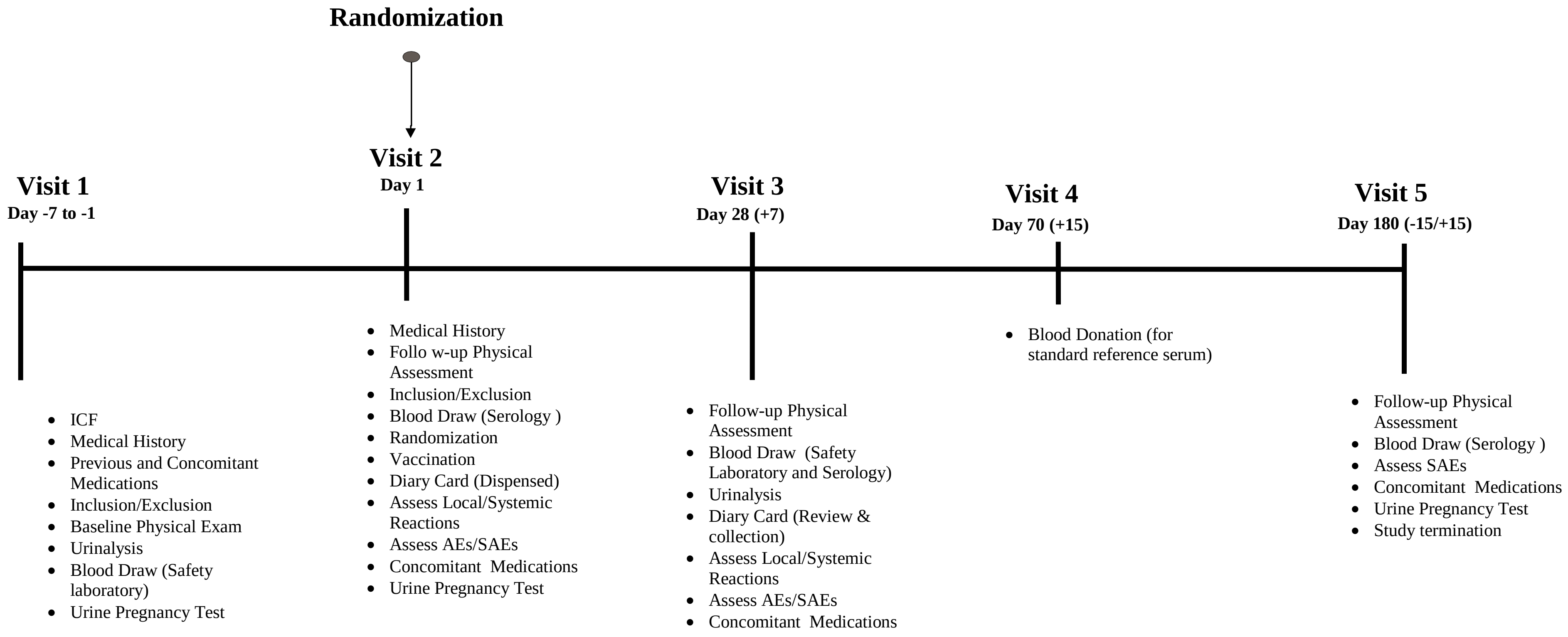

STUDY PROCEDURES:

The study is divided into four periods: Screening, vaccination, immediate and long term post vaccination follow up periods.

Screening

Visit 1: Day -7 to Day -1

Screening will be performed -7 to -1 day prior vaccination. The following procedures will be performed:

- Study procedures should be explained and informed consent must be obtained from the subject prior to any study related procedures.

- A screening number is assigned.

- Record medical history of the subject. The medical history should include details of any significant present and past diagnosis in the last 12 months (for details refer to table 6.2.1).

- Perform complete physical examination of the subject including but not limited to: blood pressure, lungs and heart. The physical examination must be performed by a qualified health professional designated within the Site Responsibility Delegation Log.

- Collect 20 mL blood for safety laboratory tests, that will include hematological and hematochemical tests (see Appendix II).

- Collect urine for urinalysis.

- Record all concomitant medications and vaccines (received within last 4 weeks), for details refer to table 6.2.1.

- Perform urine pregnancy test and confirm that the female subject is not pregnant.

- Indicate that subjects of child-bearing potential must practice appropriate birth control from this visit until study conclusion.
    - Female of childbearing potential is defined as a pre-menopausal female capable of becoming pregnant. This does not include females who meet any of the following conditions: (1) menopause at least 2 years earlier, (2) tubal ligation at least 1 year earlier, or (3) total hysterectomy.
    - Reliable birth control method is defined as hormonal (e.g., oral, injection, transdermal patch, implant, cervical ring), barrier (e.g., condom with spermicide or diaphragm with spermicide), intrauterine device (e.g., IUD),

or monogamous relationship with partner who has been vasectomized for 6 months or more prior to the subject's study entry.

- Confirm that the subject meets ALL inclusion and NO exclusion criteria and schedule subject for next visit for vaccination.

Vaccination

Visit 2: Day 1 (injection)

- Obtain significant medical history since last visit including new injuries, hospitalization, major surgeries, and any significant medical conditions which may impair the assessment of immunogenicity or safety of the vaccine.

- Perform physical examination directed by medical history, including inspection of skin over intended vaccination site and a check of general physical appearance. The physical examination must be performed by a qualified health professional designated within the Site Responsibility Delegation Log.
  Perform assessment of subject's axillary temperature.
  Note: If axillary temperature is ≥38.0 °C just prior to vaccination when measured using a validated device, vaccination must be postponed until the fever has resolved.

- Record all concomitant medications and vaccines (received since last visit), for details refer to table 6.2.1.

- Perform urine pregnancy test and confirm that the subject is not pregnant.

- If the subject continues to meet all eligibility criteria, **randomize** the subject by assigning the subject number.

- Collect 10 mL blood for serology before vaccine administration.

- Administer the vaccine according to the study group assigned by randomization. The injection will be administered by an unblinded study staff.

- During the 2 hours following injection, observe the subjects. At the end of the observation period assess for Local/Systemic Reactions.

- Dispense the diary card to the subject and explain in detail instructions for completion. Instruct the subject in how to record axillary temperature, report local and systemic reactions, AEs (Adverse Events), concomitant medications (prescription and non prescription). Also instruct the subject to notify the site immediately if they experience any SAEs (Serious aAdverse Events) or event of concern to them.

- Confirm birth control measures and remind that subjects of child bearing potential must practice appropriate birth control from this visit until study conclusion.

- Schedule the subject for Visit 3 (short-term follow-up visit).

Post-vaccination Follow Ups

Visit 3: Day 28 (window +7)

- Assess any AEs or SAEs since last visit.

- Perform the physical examination directed by medical history. The physical examination must be performed by a qualified health professional designated within the Site Responsibility Delegation Log.

- Record all concomitant medications administered as treatment of AE and all vaccines – excluding study vaccines (received since last visit).

  If the subject received concomitant medications not allowed in the study and/or vaccines, the protocol violation should be reported

- Collect subject's Diary Card. Review Diary Card with the subject for completeness, especially regarding descriptions of any local or systemic reactions, AEs or concomitant medication use. Record information obtained on appropriate source documents and CRF pages.

- Collect 20 mL blood for safety laboratory tests, that will include hematological and hematochemical tests (see Appendix II).

- Collect 10 mL blood for serology.

- Collect urine for urinalysis.

- Confirm birth control method and remind that subjects of child bearing potential must practice appropriate birth control from this visit until study conclusion.

- Discuss the window for the possible blood donation visit (Visit 4) with the subject.

- Schedule the subject for Visit 5.

Visit 4: Day 70 (window +15)

From selected volunteers of the two study groups with high antibody titers, collect a blood sample of up to 100 ml for the purpose of creating a standard reference serum and standardizing the S. Typhi serological assay.

Visit 5: Day 180 (window ±15)

- Obtain relevant medical history to assess SAEs since last visit.
- Perform physical examination. The physical examination must be performed by a qualified health professional designated within the Site Responsibility Delegation Log.
- Record all concomitant medications administered as treatment of SAE
- Collect 10 mL blood for serology.
- Inform the subject of study termination and complete Study Termination CRF.

3.3.1 Subject Numbering

A **screening number** will be given after a subject signs the informed consent form during Visit 1. The screening number will be recorded on the Screening Log. In the event the subject is not eligible and is considered a screen failure, the reason for screen failure must be documented in the Screening Log.

A screened subject will be identified by a 6-digit screening number. The first 3 digits will be 910 (corresponding to the 9 plus investigator site number) and the last 3 digits identifying the subject within the site. The digits identifying the subject within the site are assigned sequentially, with 001 corresponding to the first subject screened at study site.

A subject who continues to meet all inclusion criteria and none of the exclusion criteria will be randomized and given a **subject number** during Visit 2. A randomized subject will be identified by a 5-digit subject number. The first 2 digits will be 10 (corresponding to the investigator site number) and the last 3 digits identifying the subject within the site. The digits identifying the subject within the site are assigned sequentially, with 001 corresponding to the first subject enrolled at study site.

Once assigned to a subject, the subject and screening number cannot be reused.

The investigator must record the names of the subjects (screened and randomized) and their identifying numbers in a Subject Identification Code List.

To each subject enrolled in the study will be assigned a **subject code**. To respect data protection, the use of the initials will be avoided. Therefore, instead of using the subject's initials, the second letter of the first name followed by the second letter of the middle name or a dash (according to the convention adopted by the site) and then by the second

letter of the last name (surname) will be used. If a subject does not have a middle name a dash should be included.

3.3.2 Method of Assignment to Study Groups

The eligible subjects will be randomized to the study group on a 1:1 ratio (see section 5.0 for vaccines composition).

Hidden Entry Envelopes (HEEs) containing vaccines group information will be used in this trial, thus preventing tampering and reading of the assigned group before the subject number is given.

Laboratories performing serology analysis will be blinded to individual participant group assignments.

3.3.3 Blinding procedures

This is a single blind study: the subjects are not informed of their treatment group assignment, but the investigator, and/or other site personnel and sponsor staff (with appropriate access rights to the trial data) will have access to the group assignment of subjects once they have been randomized.

The HEEs will be produced by the BCDM department using a system that automates the random assignment of treatment arms to subject numbers with a 1:1 allocation ratio. Designated unblinded personnel at the BCDM department will be responsible to the production of the randomization list and the HEEs. The HEEs will be delivered to the study site prior to the initiation of the study.

HEE will allow revealing the vaccination group assignment on a per-subject basis; they must be stored in a secure place and opened one by one, only after the subject has been found eligible to be enrolled.

3.3.4 Vaccine Supply, Storage, Tracking and Labeling

Novartis Vaccines Institute for Global Health will supply the vaccines to the investigational site. Temperature will be monitored during the shipment. The investigator should acknowledge receipt of the study vaccines. Upon receipt, investigator or designee should ensure study vaccines are received in good condition. The investigator shall inform immediately the sponsors of any shipment temperature out of range.

The vaccines at the site must not be used before the appropriate shipping conditions have been checked and confirmed. Study vaccine will be labeled and will comply with the legal requirements of Belgium and international guidelines. Study vaccines must be handled properly and stored in a secure location to which only the investigator or designee have access.

All study vaccines must be stored in a safe, locked, and secure place with no access by unauthorized personnel. They must be kept in the refrigerator (+2°C to +8°C) and **must not be frozen**. Storage temperature should be monitored every day. Access to a back-up refrigerator in case of power failure/breakdown is necessary.

Vaccines that have been stored differently from the sponsor's recommendations **must not** be used unless the sponsor provides written authorization for use. In the event that the use cannot be authorized, vaccine supply must be replaced with fresh stock supplied by the sponsor.

The investigator should ensure that the vaccines delivered to the site are used only in accordance with the approved protocol. Monitoring of vaccine accountability will be performed by the study monitor during site visits.

The investigator should maintain an accurate record of products delivery to the site, the inventory at the site, the administration to the subjects, and the return to the sponsor, or destruction, of study vaccines. At the conclusion, and as appropriate during the course of the study, the investigator will return to the sponsor, or destroy at study site (as per sponsor requirements and SOP) all used and unused study vaccines, packaging and supplementary labels. If the unused study vaccines are disposed at the site, the investigator should provide a copy of the site's procedure for destruction of hazardous material and documentation of the destruction.

Study vaccines will be labeled to comply with the legal requirements of Belgium and international guidelines. All study vaccines must be stored according to the instructions specified on the labels.

3.3.5 Processing, Labeling and Storage of Serum Samples for Serology

For measuring anti-Vi antibody titers (ELISA) in the serum, 10 mL of blood will collected from each subject at visits 2 (pre vaccination), 3 (post vaccination) and 5 (persistence post vaccination). Additional serology tests to evaluate the immunogenicity of the study vaccines may be performed if deemed necessary by the sponsor.

Blood samples must be collected in the appropriate manner, using exclusively materials and guidelines supplied by the sponsor. The investigator must ensure that his/her personnel and the laboratory(ies) under his/her supervision comply with this requirement.

Serum samples will be stored frozen at -20ºC. Shipment to the different laboratories for analysis will be performed according to sites guidelines provided by the sponsor.

Complete instructions for processing, labeling, storage and shipping of samples are included in the Serology Guidelines.

3.4 Duration of Subject's Expected Participation in the Entire Study

Enrollment period of the study is expected to be 5 days. Each subject is expected to participate to the study for up to 187 days, including up to 7 days of screening period, and 180 days follow up period.

3.5 Stopping/Pausing Rules

There are no predetermined stopping rules. However, in case a SUSAR will occur or in case of increased rate of SAE, the sponsor will assess the overall safety profile.

The sponsor or the investigator (following consultation with the sponsor) have the right to discontinue this study at any time. If the clinical study is prematurely terminated, the investigator must promptly inform the study subjects and the EC and should assure appropriate follow-up for the subjects. All procedures and requirements pertaining to the archiving of the documents should be followed. All remaining study materials must be returned to the sponsor.

4.0 SELECTION OF STUDY POPULATION

4.1 Inclusion Criteria

1. Males and females of age ≥18 to ≤40 years.
2. Individuals who, after the nature of the study has been explained to them, have given written consent according to local regulatory requirements.
3. Individuals in good health as determined by the outcome of medical history, physical examination, hematological / hematochemical blood tests and urinalysis and clinical judgment of the investigator.
4. If women, a negative pregnancy test and willing to use birth control measures for the entire study duration.

4.2 Exclusion Criteria

1. Individuals with behavioral or cognitive impairment or psychiatric disease that, in the opinion of the investigator, may interfere with the subject's ability to participate in the study.
2. Individuals with any progressive or severe neurological disorder, seizure disorder or Guillain-Barré syndrome.
3. Individuals who are not able to understand and to follow all required study procedures for the whole period of the study.

4. Individuals with history of any illness that, in the opinion of the investigator, might interfere with the results of the study or pose additional risk to the subjects due to participation in the study.

5. Individuals with known or suspected HIV infection or HIV related disease, with history of an autoimmune disorder or any other known or suspected impairment /alteration of the immune system, or under immunosuppressive therapy including use of systemic corticosteroids or chronic use of inhaled high-potency corticosteroids within the previous 30 days, or were in chemotherapy treatment within the past 6 months.

6. Individuals with a known bleeding diathesis, or any condition that may be associated with a prolonged bleeding time.

7. Individuals with any serious chronic or progressive disease according to judgment of the investigator (neoplasm, insulin dependent diabetes, cardiac, renal or hepatic disease).

8. Individuals who have any malignancy or lymphoproliferative disorder.

9. Individuals with history of allergy to vaccine components.

10. Individuals participating in any clinical trial with another investigational product 30 days prior to first study visit or intent to participate in another clinical study at any time during the conduct of this study.

11. Individuals who have previously received any vaccines against typhoid fever (either oral live attenuated or injectable vaccines).

12. Individuals who received any other vaccines within 4 weeks prior to enrollment in this study or who are planning to receive any vaccine within 4 weeks from the study vaccine.

13. Individuals who have received blood, blood products and/or plasma derivatives including parenteral immunoglobulin preparations in the past 12 weeks.

14. Individuals who are part of study personnel or close family members conducting this study.

15. Individuals with body temperature $\geq$ 38.0 degrees Celsius within 3 days of intended study immunization.

16. BMI > 35 kg/m^2.

17. Individuals with history of substance or alcohol abuse within the past 2 years.

18. Women who are pregnant or breast-feeding or of childbearing age who have not used or do not plan to use acceptable birth control measures, for the duration of the study.

19. Females with history of stillbirth, neonatal loss, or previous infant with anomaly.

20. Individuals who have a previously ascertained or suspected disease caused by S. Typhi.

21. Individuals who have had household contact with/and or intimate exposure to an individual with laboratory confirmed S. Typhi.

22. Any condition which, in the opinion of the investigator may interfere with the evaluation of the study objectives.

4.3 Withdrawal of Subjects from Therapy or Assessment

The subject can withdraw consent for participation in the study at any time without penalty or loss of benefit to which the subject is otherwise entitled. The investigator can withdraw a subject if, in his or her clinical judgment, it is in the best interest of the subject or if the subject cannot or will not comply with the protocol.

If a subject withdraws from the study, the reason for withdrawal should be documented in the subject's medical record and reported in CRF.

<u>For women of childbearing potential</u>:

Any subject, who, despite the requirement for adequate contraception, becomes pregnant during the trial, will be followed-up. The site should maintain contact with the pregnant subject, complete a "Pregnancy Report" CRF as soon as possible, and obtain pregnancy outcome information for "Pregnancy Follow-up" CRF. In case of withdrawal, the subject should be followed up and the reason for withdrawal (e.g. pregnancy) should be recorded in detail on the "Study Termination" CRF as well as on the subject's medical records (see section 6.2.5 for further details).

Withdrawn subjects will not be replaced.

5.0 TREATMENT OF SUBJECTS

Subjects will be randomly assigned in a 1:1 ratio, to receive one of the following vaccines:

- 0.5 mL dose of NVGH Vi-CRM_{197} vaccine
- 0.5 mL dose of licenced Vi polysaccharide vaccine (Typherix®,GlaxoSmithKline)

All study vaccines are to be kept in a secure location with appropriate storage conditions, temperature monitoring, and separate from other vaccines.

5.1 Investigational Vaccine - NVGH S. Typhi conjugate vaccine

The Vi-CRM_{197} vaccine contains the purified Vi polysaccharide derived from Citrobacter ------------. The Vi polysaccharide is chemically conjugated to CRM_{197}, the mutant diphtheria toxin carrier protein.

Vi-CRM_{197} is prepared in an approximate 1:1 ratio by weight of polysaccharide to protein. Each conjugate molecule is estimated to contain 3-4 protein chains and a single saccharide moiety.

The vaccine is provided in the form of one vial containing a fully liquid Vi-CRM_{197}. The volume of the Vi-CRM_{197} conjugate vaccine contained in the vial is approximately 0.7 mL out of which 0.5 mL should be used as immunizing dose in the deltoid of the non-dominant arm.

The product is ready for intramuscular injection. A 0.5 mL dose of NVGH Vi vaccine contains: 25 µg of Vi; 25 µg CRM_{197} and 29 mM of Sodium phosphate buffer.

For additional information please refer to the investigator brochure provided.

5.2 Control Vaccine – GSK Typherix®

The licensed Vi polysaccharide vaccine (Typherix® ,GlaxoSmithKline) is a clear isotonic colourless solution.

Vi antigen is extracted from the bacterial capsule of S. Typhi strain TY2. A 0.5 mL dose of Vi polysaccharide vaccine contains 25 µg of Vi. One 0.5 mL dose out of single dose pre-filled syringes of Vi polysaccharide vaccine will be injected IM in the deltoid of the non-dominant arm.

For additional information please refer to the vaccine package insert provided.

5.3 Concomitant Vaccines or Treatment

No concomitant vaccines will be studied as part of this trial.

No concomitant vaccines are allowed during the 4 weeks period before and after vaccination (Visit 2). Subjects who received any other vaccines within 4 weeks prior to study vaccination or who are planning to receive any vaccine within 4 weeks after are excluded from the study (see exclusion criteria #12).

5.4 Vaccines Preparation and Administration

The principal investigator or designee will be responsible for the administration of the vaccine to subjects enrolled into the study according to the procedures stipulated in this

study protocol. All vaccines will be administered only by personnel who is qualified to perform that function under applicable local laws and regulations for the specific study site.

One 0.5 mL dose out of a single dose vial of vaccine will be injected intramuscularly in the deltoid of the non-dominant arm. The vaccine must be kept at room temperature few minutes before drawing into a syringe with a 25 mm needle.

The licensed vaccine must be prepared according to the package insert before use.

PRECAUTIONS TO BE OBSERVED IN ADMINISTERING STUDY VACCINE:

The vaccination site should be disinfected with a skin disinfectant (e.g., 70% alcohol). Before vaccination, the skin must be dry. **DO NOT inject intravascularly**.

Standard immunization practices should be observed and care should be taken to administer the injection intramuscularly. As with all injectable vaccines, appropriate medical treatment and supervision should be readily available (medication available in the room where the vaccines are administered), in case of anaphylactic reactions within the 2 hours observation period following administration of the study vaccine. According to the Belgian guidelines (2004) from the Higher Health Council, epinephrine 1:1000 should be available in case of any anaphylactic reactions.

Study vaccines should not be administered to individuals with known hypersensitivity to any component of the vaccines.

Vaccinations must not be administered to any subject with a clinically significant active infection (as assessed by the investigator) or measured by body (axillary) temperature ≥ 38.0°C/100.4ºF within 3 days of the intended date of vaccination. If either of these is observed, vaccination should be postponed until the subject's temperature remains below 38.0°C/100.4°F for at least 3 days or the investigator feels that the subject's illness has stabilized, as appropriate.

5.5 Other Concomitant Treatment or Vaccines

The following medications will be recorded in CRF:

- Prescription and non prescription medications taken by the subject for the treatment of any SAEs or AEs within 28 days (up to Visit 3) after Visit 2
- All vaccinations received by the subject within 28 days (up to Visit 3) after Visit 2
- Immunosoppressants or other immune-modifying drugs, investigational drugs/vaccines, immunoglobulins or any blood products, as well as any medications taken by subject as treatment for SAEs from Visit 3 to the end of study (up to Visit 5).

Although protocol violations to be treated as such, also the following medications will have to be recorded in the CRF if they occur:

- Investigational products taken by the subject within 4 weeks prior to Visit 2
- Vaccines received by the subject within 4 weeks prior to Visit 2
- Blood, blood products and/or plasma derivates including parenteral immunoglobulin preparations taken by the subject within 12 weeks prior to Visit 2

Prior medications include (at a minimum) all prescription medications taken regularly by a subject at the time of study enrollment.

Use of the following concomitant medications after enrollment may interfere with the interpretation of the study objectives or indicate an underlying condition resulting in a major protocol violation according to the medical judgment of the investigator and NVGH physician (Dr. Audino Podda, phone:+39 0577 243496):

- Systemic Corticosteroids
- Blood or blood products

5.6 Vaccination Compliance

The investigator is responsible for adequate and accurate accounting of vaccine usage. The investigator or designee will administer the study vaccines only to individuals included in this study following the procedures set out in this study protocol. The date, dosage, and time of the vaccinations must be recorded. The investigator must track vaccines received, used and wasted and will retain all unused or expired products as described in section 3.3.4.

6.0 IMMUNOGENICITY AND SAFETY ASSESSMENTS

6.1 Appropriateness of Measurements

For immunogenicity: The measures of immunogenicity used in this study are standard, i.e., widely used and generally recognized as reliable, accurate, and relevant (able to describe the quality and extent of the immune response).

For safety: The measures of safety used in this study are routine clinical and laboratory procedures. They include a close vigilance for, and stringent reporting of, selected local and systemic reactions routinely monitored in vaccine clinical trials as indicators of reactogenicity.

6.1.1 Immunogenicity

The primary measure of immune response is the anti-Vi ELISA antibody titer obtained 28 days after vaccination (Visit 3, Day 28).

6.1.2 Methods, Criteria and Timing for Assessing and Recording Efficacy/Immunogenicity Parameters

Blood samples for immunogenicity testing are obtained at Days 1, 28 and 180. The primary measure of immune response is the anti-Vi ELISA antibody titer obtained at Day 28 (28 days after the injection).

6.2 Safety Parameters

At each study visit, the Investigator should question the subject about any medication taken. Investigator will record medications (as instructed in Table 6.2.1) in the CRF with trade name and/or generic name, medical indication, total daily dose, route of administration, start and end dates of treatment.

Table 6.2.1: Medical and Safety Assessments to be reported into CRF

**Before Visit 2 vaccination**	**Medical History:**<br>Any significant past diagnoses in the last 12 months including injuries, hospitalizations, major surgeries, or other significant medical conditions which may impair the assessment of immunogenicity or safety of the study vaccine
	**Medications:**<br>All vaccinations received in the 4 weeks before study vaccination.<br>All investigational products received during the previous 30 days<br>Blood , blood products and /or plasma derivates including parenteral immunoglobulin preparations during the previous 12 weeks
**During 2 hours after vaccination**	**Immediate reaction:**<br>Signs or symptoms of anaphylaxis
	**Local reactions:**<br>Pain, Erythema, Induration
	**Systemic reactions:**<br>Chills, Malaise, Myalgia, Headache, Arthralgia, Fatigue
**During 7 days after vaccination (Day 1 to Day 7)**	**Temperature:**<br>Axillary temperature (Fever is defined as axillary temperature ≥ 38.0 °C),
	**Local reactions:**<br>Pain, Erythema, Induration
	**Systemic reactions:**<br>Chills, Malaise, Myalgia, Headache, Arthralgia, Fatigue
**From Visit 2 to Visit 3**	**All Adverse Events:**<br>All AEs will be monitored until resolution or, if the AE becomes chronic, a cause identified. If an AE is unresolved at the conclusion of the study, a clinical assessment will be made by the investigator

	and medical monitor whether continued follow-up of the AE is warranted.
	**Medications (all subjects):**<br>Any concomitant medications administered as treatment of AE and all vaccinations (excluding the study vaccination).
**All study (for 180 days)**	**All Serious Adverse Events:**<br>All SAEs must be documented and followed up until the event is resolved, subsided, stabilized, disappeared or is otherwise explained or the subject is lost to follow-up. *If SAE remains unresolved at study termination, a clinical assessment will be made by the investigator and the sponsor Medical Monitor to determine whether continued follow up of the SAE is warranted.*<br><br>Any SAE occurring at any time outside the 180 days study period and considered to be caused by the study vaccine will be reported.
	**Medications:**<br>Any medications administered as treatment of SAE, immunosuppressants or other immune-modifying drugs, investigational drugs/vaccines, immunoglobulins or any blood products.

A brief medical history will be obtained and physical examination performed for each subject entered into the study.

Solicited reactogenicity, including local and systemic reactions and other unsolicited adverse events will be collected in the study, as detailed in sections 6.2.1 to 6.2.5 and Table 6.2.1.

Reactogenicity will be assessed daily during the first week following vaccination: local and systemic reactions occurring at day 1 to day 7 will be recorded in the diary card. The overall safety follow-up will be 6 months (180 days) for each subject: all adverse events (AE) will be collected and documented for 28 days; serious adverse events (SAE) will be collected and documented for the duration of the study (180 days).

6.2.1 Local and Systemic Reactions

The occurrence of selected indicators of reactogenicity (listed in Table 6.2.1) and other unsolicited adverse events will be recorded in the diary card by the subject and reported in the corresponding CRF.

6.2.2 Adverse Events

An adverse event (AE) is defined as any untoward medical occurrence in a subject or clinical investigation subject administered a pharmaceutical product at any dose that does not necessarily have to have a causal relationship with this treatment. An AE can, therefore be any unfavorable and unintended sign (including an abnormal laboratory

finding, for example), symptom, or disease temporally associated with the use of an investigational product, whether or not considered related to the investigational product. This definition includes intercurrent illnesses or injuries and exacerbation of pre-existing conditions.

An unexpected adverse event is one that is not listed in the current Summary of Product Characteristics or the Investigator's Brochure or an event that is by nature more specific or more severe than a listed event.

All AEs will be monitored until resolution or, if the AE becomes chronic, a cause identified. If an AE is unresolved at the conclusion of the study, a clinical assessment will be made by the investigator and medical monitor whether continued follow-up of the AE is warranted.

The severity of events reported on the "Adverse Events" CRF will be determined by the investigator as:

Mild:	transient with no limitation in normal daily activity.
Moderate:	some limitation in normal daily activity.
Severe:	unable to perform normal daily activity.

The relationship of the study treatment to an AE will be determined by the investigator based on the following definitions:

1. Not Related

The AE is not related to a investigational vaccine if there is evidence that clearly indicates an alternative explanation. If the subject has not received the vaccine, the timing of the exposure to the vaccine and the onset of the AE are not reasonably related in time, or other facts, evidence or arguments exist that strongly suggest an alternative explanation, then the AE is not related.

2. Possibly Related

The administration of the investigational vaccine and AE are considered reasonably related in time **and** the AE could be explained either by exposure to the investigational vaccine or by other causes.

3. Probably Related

Exposure to the investigational vaccine and AE are reasonably related in time and no alternative explanation has been identified.

The relationship of the study treatment to an AE will be determined by the investigator.

6.2.3 Serious Adverse Events

A serious adverse event (SAE) is defined as any untoward medical occurrence that at any dose:

- Results in death.
- Is life-threatening (i.e., the subject was, in the opinion of the investigator, at immediate risk of death from the event as it occurred); it does not refer to an event which hypothetically might have caused death if it were more severe.
- Requires or prolongs subject’s hospitalization.
- Results in persistent or significant disability/incapacity (i.e., the event causes a substantial disruption of a person’s ability to conduct normal life functions).
- Results in a congenital anomaly/birth defect.
- Requires intervention to prevent permanent impairment or damage.
- Is an important and significant medical event that may not be immediately life threatening or resulting in death or hospitalization but, based upon appropriate medical judgment, may jeopardize the subject or may require intervention to prevent one of the other outcomes listed above.

Grade 4 and 5 laboratory deviations are also considered SAEs.

Of note: a “possible vaccine failure” should be reported as a SAE only if it resulted in a laboratory confirmed infectious disease which should have been prevented by the vaccine implied.

Adverse events which do not fall into these categories are defined as **non-serious**.

It should be noted that a severe adverse event need not be serious in nature and that a serious adverse event need not, by definition, be severe.

In addition, a pre-existing event or condition that results in hospitalization should be recorded on the Medical History CRF. If the onset of an event occurred before the subject entered the trial (e.g., any pre-planned hospitalization for conditions like cosmetic treatments or for non-emergency routine visits for a pre-existing condition), the hospitalization would not lead to an AE being classified as serious unless, in the view of the investigator, hospitalization was prolonged as a result of participation in the clinical trial or was necessary due to a worsening of the pre-existing condition.

For instructions on SAE reporting, please refer to section 6.2.6.

6.2.4 Subject's Diary Card

Diary Cards will be completed by the subject and used by the study staff to record safety information (concomitant medications and vaccinations, local and systemic reaction, AE and SAE) occurring in the 28 days after vaccination (up to study Visit 3).

According to ICH GCP guidelines 1.52, subject's Diary Card is a "Source Document". It is an investigator responsibility to ensure that data on the diary card correspond to the real health status of subject and to accurately transcribe data from the Diary Card to the CRF.

In case the Diary Card is not available, the study staff will document the reasons for the missing documents on the Comment section of the CRF and the CRF will be completed with UNK for all assessments. Nevertheless, if a SAE occurred it must be recorded in SAE form and in the adverse event section of CRF.

6.2.5 Pregnancies

To ensure subjects' safety, each pregnancy in a subject on study vaccine must be reported to Novartis Vaccines Institute for Global Health within 24 hours of learning of its occurrence. The pregnancy should be actively followed up to determine outcome, including spontaneous or voluntary termination, details of the birth, and the presence or absence of any birth defects, congenital abnormalities, or maternal and/or newborn complications.

Any SAE experienced during pregnancy must be reported on the SAE Report Form.

6.2.6 Methods and Timing for Assessing and Recording Safety Parameters

The period of observation for adverse events starts from the time the subject receives the vaccination. All study subjects will be observed at Site for at least 2 hours after vaccination for evidence of immediate reactions and in particular for symptoms of allergic phenomena (such as rashes or other allergic manifestations). Each subject will be instructed to complete a diary card for 28 days following vaccination, to describe local and systemic reactions (during the 7-day period after vaccination) and AE (during the 28-day period after vaccination). After visit 3, all Serious Adverse Events (SAEs) will be collected and documented.

All adverse events occurring within the above period, regardless of severity, will be monitored by the investigator until resolution. All subjects experiencing adverse events - whether considered associated with the use of the study vaccine or not - must be monitored until symptoms subside and any abnormal laboratory values have returned to baseline, or until there is a satisfactory explanation for the changes observed, or until death, in which case a full pathologist's report should be supplied, if possible. All findings must be reported on an "Adverse Events" CRF and on the "Vaccine Serious

Adverse Event" form, if necessary. All findings in subjects experiencing adverse events must be reported also in the subject's medical records.

All SAEs which occur during the course of the trial, whether considered to be associated with the study vaccination or not, must be reported **within 24 hours** or at the latest on the following working day by telephone or fax to Novartis Vaccines Institute for Global Health. The "Vaccine Serious Adverse Event" form is to be completed for all SAEs and faxed to the sponsor.

Study Contacts for Reporting Serious Adverse Events

Audino Podda, MD
Novartis Vaccines Institute for Global Health (NVGH)
Via Fiorentina, 1 - 53100 Siena, Italy
Phone:+39 0577 243496
Fax: +39 0577 539114
Mobile: +39 335 7026950
Email: audino.podda@novartis.com

The original form is retained by the investigator. The event is also documented on the "Adverse Events" CRF. After receipt of the initial report, the Physician/site CRA will review the information and contact the investigator if it is necessary to obtain further information for assessment of the event. Any medication or other therapeutic measures used to treat the event will be recorded on the appropriate CRF(s) in addition to the outcome of the AE. Any serious adverse reaction must be reported to the EC or IRB by the Site in a timely manner. Adequate documentation will be provided to the sponsor showing that the EC or IRB has been properly notified. The sponsor must also comply with the applicable regulatory requirement(s) related to the reporting of unexpected serious and non-serious adverse vaccine reactions to the regulatory authority(ies) and the IRB/IEC.

If required, a follow-up report including all new information obtained on the serious adverse event must be prepared and sent to NVGH. The report should be marked "Follow-up report."

The investigator will submit, on request, copies of all these reports to the EC or IRB and other relevant authorities.

Post-Study Events

Any SAE occurring at any time after the end of the study and considered to be caused by the study vaccine - and therefore a possible adverse reaction - must be reported by using a SAE Report Form to:

Audino Podda, MD

Novartis Vaccines Institute for Global Health (NVGH)
Via Fiorentina, 1 - 53100 Siena, Italy
Phone:+39 0577 243496
Fax: +39 0577 539114
Mobile: +39 335 7026950
Email: audino.podda@novartis.com.

6.4 Data Safety Monitoring Board

A Data Safety Monitoring Board (DSMB) will not be convened for this study.

7.0 STATISTICAL PLAN

7.1 Statistical Hypothesis

This Phase I safety and immunogenicity trial is aimed to descriptively evaluate the safety and immunogenicity profiles of the study vaccines. No specific hypotheses are tested in this trial.

7.2 Sample Size and Power Considerations

Given the sample size selected for this trial, the precision to define certain incidence rates and the power to detect differences in the incidence rates between the vaccine groups in ad hoc or exploratory analyses will be indicated in the following examples:

- For each vaccine, with 25 subjects per vaccine group, there is a 90% probability of observing at least 1 subject with an adverse event if the true rate of such an event is 8.7%.

  The probability to detect at least one subject with an adverse event (for each of the vaccine groups) is reported in Table 1 by underlying rates of AEs descending from 15% - 1.5%:

Table 1

Underlying Event Rate (Frequency of AE)	Probability of Detection N=25
15%	98%
12.5%	96%
10%	93%
7.5%	86%
5%	72%
2.5%	47%

1.5%	31%

- Comparative statistics for the safety variables will not be calculated, the study has low power to detect anything other than large differences in the incidence of local and systemic reactions between vaccination groups. In fact, a difference between a 10% reaction rate (n=25) in one of the vaccine groups and a 20% reaction rate in one of the other vaccine groups (n=25) is detectable with only 8% power at a 5% two-sided alpha level. Safety comparisons, with an 80% power and a 5% two-sided alpha level, will permit to detect differences (calculated by using the Fisher exact test) as reported in Table 2:

Table 2

**Underlying Event Rate in one vaccine group**	**Difference detected with a 80% probability**
30%	41.0%
20%	40.7%
10%	38.5%
5%	34.0%
2.5%	31.5%

- The precision of the estimates, expressed by the 95% Clopper-Pearson CI, for several possible observed values of the percentages of seroprotection and seroconversion for all vaccine groups is shown in Table 3:

Table 3

**Response Rate**<br>**%**	**95% CI**<br>**(N = 25)**<br>**% %**
48	27.8 - 68.7
52	31.3 - 72.2
56	34.9 - 75.6
60	38.7 - 78.9
64	42.5 - 82.0
68	46.5 - 85.1
72	50.6 - 87.9
76	54.9 - 90.6
80	59.3 - 93.2
84	63.9 - 95.5
88	68.8 - 97.5
92	74.0 - 99.0
96	79.6 - 99.9
100	86.3 - 100.0

- In performing an immunogenicity comparison, a sample sizes of 25 per group will permit to detect a 2.5-fold difference in antibody titer with 93% power at alpha=0.05 when the standard deviation of $\log_{10}$ titers is no greater than 0.4.

7.3 Population for Analysis

Definition of populations to be analyzed:

a) All Randomized Population

- all subjects who:
  - signed informed consent
  - are randomized into the study.

  This population will be used for the analysis of demographics and all subject listings.

b) Immunogenicity Population -- Modified Intention-to-Treat (mITT) population

- all subjects in the randomized population who:
  - actually receive study vaccination, and
  - provide at least one evaluable serum sample at Visit 2 or Visit 3.

c) Immunogenicity Population -- Per Protocol (PP) population

- all subjects in the mITT Immunogenicity population who:
  - receive the correct vaccine as assigned in the randomization list
  - provide evaluable serum samples at both Visits 2 and 3, and
  - have no major protocol violation as defined prior to unblinding.

A major deviation is defined as a protocol deviation that is considered to have significant impact on the immunogenicity result of the subject.

d) Safety population

- all randomized subjects who:
  - receive at least one study vaccine
  - provide post-vaccination safety data

7.4 Analysis of Demographic and Baseline Characteristics

Descriptive statistics (mean, standard deviation, median, minimum and maximum) for age, height and weight at enrollment will be calculated overall and by vaccine group.

Distributions of subjects by sex and ethnic origin will be summarized overall and by vaccine group.

7.5 Analysis of Efficacy/Immunogenicity Endpoints

7.5.1 Description of Response Variables

The primary response variable is the Vi specific nAb (neutralizing antibody) level measured 28 days after the vaccination.

7.5.2 Statistical Methods for Efficacy/Immunogenicity Variables

The measures of immunogenicity, against the Vi antigen of S.Typhi, will include:

- Geometric mean concentrations (GMCs), pre- and post-vaccination, as determined by ELISA, and applicable geometric mean ratios between post- and pre-vaccination samples.

- Seroconversion rate: percentage of subjects achieving at least a four-fold rise in ELISA antibody titer in the post-vaccination blood sample

- Seroprotection rate: percentage of subjects achieving an ELISA titer ≥ 7 units, the estimated threshold of protection[4], in the post-vaccination blood sample.

7.6 Analysis of Safety (Endpoints) and Tolerability

The measures of safety will include:

- Deviations from normal values (see Appendix II) of hematological and haematochemical blood tests and urinalysis after immunization. Clinical significance of abnormal values may be assessed by medical judgment.

- Numbers and percentage of subjects with solicited local and systemic adverse reactions as well as numbers and percentage of subjects with reported unsolicited adverse events and serious adverse events.

Solicited local reactions include erythema, induration and pain at injection site; solicited systemic reactions include headache, arthralgia, chills, fatigue, malaise, myalgia, and fever as measured by axillary temperature for Day 1 through 7 of the study.

All local and systemic reactions will be collected for 7 days after immunization (i.e., days 1 to 7). All AEs will be collected for 28 days and all SAEs will be collected throughout the entire study (180 days).

All SAEs and AEs (including onset of chronic illness) will be judged by the Investigator as either probably related, possibly related, or not related to vaccine and will be tabulated. All SAEs and AEs resulting in withdrawal from the study will be summarized.

7.6.1 Analysis of Extent of Exposure

Not applicable.

7.6.2 Analysis of Local and Systemic Reactions

Frequencies and percentages of subjects experiencing each reaction will be presented for each symptom severity. Summary tables showing the occurrence of any local or systemic reaction overall and at each time point will also be presented.

Post-vaccination reactions reported from day 1 to day 7 will be summarized by maximal severity and by vaccine group. The severity of local reactions, including injection-site erythema and induration will be categorized as none, 1 to $\leq$10 mm, 11 to $\leq$25 mm, 26 to $\leq$50mm, 51 to $\leq$100 mm and >100 mm (severe local reactions).

The severity of pain and systemic reactions (e.g., chills, malaise, myalgia, headache, etc. as per section 6.2.1) occurring up to 7 days after vaccination will be categorized as none, mild (transient with no limitation in normal daily activity), moderate (some limitation in normal daily activity), and severe (unable to perform normal daily activity).

Axillary temperature will be categorized as <38°C (no fever), 38-38.9°C (mild), 39-39.9°C (moderate) and ≥40°C (severe).

No statistical inference will be performed for the local and systemic reaction safety variables.

7.6.3 Analysis of Other Adverse Events

All the adverse events occurring during the study, judged either as related to vaccination or not by the investigator, will be recorded as specified in section 6.2.5. The original verbatim terms used by investigators to identify adverse events in the CRFs will be mapped to preferred terms using the MedDRA dictionary. The adverse events will then be grouped by MedDRA preferred terms into frequency tables according to system organ class. All reported adverse events, as well as adverse events judged by the investigator as at least possibly related to study vaccine, will be summarized according to system organ class and preferred term within system organ class. These summaries will be presented by vaccination group. When an adverse event occurs more than once for a subject, the maximal severity and strongest relationship to the vaccine group will be counted. Additionally, three separate summaries will be produced: (i) serious adverse events, (ii) adverse events that are possibly or probably related to vaccine, and (iii) adverse events that are unrelated to vaccine. Data listings of all adverse events will be provided by subject. In addition, a listing of subjects withdrawn from the study because of an adverse event will be presented.

7.7 Planned Interim Analysis

Preliminary analyses are planned after Day 28 data become available to evaluate safety and immunogenicity at 4 weeks after immunization in order to better plan the subsequent trials of the clinical development plan.

Individual subject results from unblinded preliminary analyses will not be made available to personnel at the clinical study sites until the end of the study.

8.0 STUDY MONITORING, AUDITING AND DOCUMENTATION

Study monitoring and auditing will be performed in accordance with the sponsor’s procedures and applicable regulatory requirements (e.g. EMEA, ICH and GCP guidelines).

Investigators and/or their study staff will be trained on the study protocol and all applicable study procedures prior to subject enrollment. CRFs supplied by the sponsor must be completed for each enrolled subject. The data entries as well as study related documents will be checked by the sponsor and/or trained delegates of the sponsor.

8.1 Study Monitoring

Study progress will be monitored by Novartis Vaccines Institute for Global Health or its representative (e.g. a contract research organization) as frequently as necessary to ensure the rights and well-being of study subjects are protected; to verify adequate, accurate and complete data collection; protocol compliance and to determine that the study is being conducted in conformance with applicable regulatory requirements. Arrangements for monitoring visits will be made in advance in accordance with the monitoring plan, except in case of emergency.

8.2 Source Data Verification

Data recorded on the eCRF will be verified by checking the CRF entries against source documents (i.e., all original records, laboratory reports, medical records, subjects diaries, memory aids) in order to ensure data completeness and accuracy as required by study protocol. The investigator and/or site staff must make CRFs and source documents of subjects enrolled in this study available for inspection by NVGH or its representative at the time of each monitoring visit.

At a minimum, source documentation must be available to substantiate subject identification, eligibility and participation, proper informed consent procedures, dates of visits, adherence to protocol procedures, adequate reporting and follow-up of adverse events, administration of concomitant medication, study vaccine receipt/dispensing/return records, study vaccine administration information, and date of completion and reason. Specific items required as source documents will be reviewed with the investigator before the study.

The source documents must also be available for inspection, verification and copying, as required by regulations, by officials of the regulatory health authorities (e.g. FDA, EMEA and others) and/or ECs/IRBs. The investigator and study site staff must comply with applicable privacy, data protection and medical confidentiality laws for use and disclosure of information related to the study and enrolled subjects.

The subject must also allow access to her medical records. Each subject should be informed of this prior to the start of the study.

9.0 DATA MANAGEMENT

<u>Electronic CRF</u>

An electronic data capture (EDC) system (e.g., Inform™) will be used to expedite the entry of data. The investigator will enter data into the web enable EDC system in a timely manner; the data will be stored in clinical database management system. eCRF data will be reviewed routinely by data management and clinical monitors.

Electronic Data Transfer (EDT) is one method being used by NVGH for collecting laboratory data. The laboratory (e.g., central laboratory) will send data as electronic files by a secured method (e.g., via diskette, CD, as an encrypted file attachment on electronic mail, or as a direct transfer into a specified server directory) to data management department. The data file is pre-processed and loaded by the data management Lab Manager into the study database. The laboratory will submit a results' file containing the tests and the results as specified in the protocol. If the laboratory provides the service, it will also submit a Demography (DEMOG) file containing the subject's demographic information. If the file includes results of data blinded to Clinical, the source will provide a separate RESULTS file that will be loaded into a separate laboratory table.

All serology data analyzed by serology laboratory will be entered into the Seroad database by the Serology Laboratory. All results will be checked in the laboratory for validity and completeness.

For this protocol, antibody laboratory data and/or safety laboratory data will be transmitted via EDT.

9.1 Data Handling Procedures

Coding will be performed using the following dictionaries:

Adverse Events:	MedDRA
Concomitant illness:	ICD-9
Concomitant and intercurrent therapy:	WHO Drug Dictionary

9.2 Documentation of Study Findings

The investigator must review and electronically sign the eCRFs to verify their accuracy. Correction to data on eCRFs will be tracked via an audit trail within InForm™, web based electronic data capture system. Each correction will be identified by the person making the change and will include time, date, and reason for change. If corrections are made to a previously and electronically signed CRF, the investigator must confirm and endorse the changes.

As part of the conduct of the trial, Novartis Vaccines Institute for Global Health may have questions about the Case Report Form data after the site has entered the data. These questions will be raised within InForm™. The Investigator will provide follow-up clarification and/or resolution of data issues raised by the monitor or the data manager.

An explanation must be provided and documented by the investigator for all missing data.

9.3 Data Protection

Novartis Vaccines Institute for Global Health respects the subjects' rights to privacy and will ensure the confidentiality of their medical information in accordance with all applicable laws and regulations.

The sponsor as Data Controller according to the European Directive on the protection of individuals with regard to the processing of personal data and on the free movement of such data [95/46/EC] confirms herewith compliance to Directive 95/46/EC in all stages of Data Management.

10.0 RECORD RETENTION

Investigators must retain all study records required by NVGH and by the applicable regulations in a secure and safe facility. The investigator must consult a NVGH representative before disposal of any study records, and must notify the sponsor of any change in the location, disposition, or custody of the study files. Essential documents must be retained until at least 2 years after the last approval of a marketing application in an ICH region and until there are no pending or contemplated marketing applications in an ICH region or at least 2 years have elapsed since the formal discontinuation of clinical development of the investigational product. "Essential documents" are defined as documents that individually and collectively permit evaluation of the conduct of a trial and the quality of the data produced. These documents should be retained for a longer period however, if required by the applicable regulatory requirements or by an agreement with the sponsor or The Committee for Human Medicinal Products fro Human Use (CHMP) requires retention for the maximum period of time permitted by the institution, but not less than 15 years (ICH E6, 4.9.5). It is the responsibility of the sponsor to inform the investigator/institution as to when these documents no longer need to be retained (ICH E6, 5.5.12).

11.0 USE OF INFORMATION AND PUBLICATION

Novartis Vaccines Institute for Global Health assures that the key design elements of this protocol will be posted in a publicly accessible database such as clinicaltrials.gov.

12.0 ETHICS

12.1 Regulatory and Ethical Compliance

This clinical study was designed and shall be implemented and reported in accordance with the ICH Harmonized Tripartite Guidelines for Good Clinical Practice, with applicable local regulations (including European Directive 2001/20/EC, US Code of Federal Regulations Title 21, and Japanese Ministry of Health, Labor, and Welfare), and with the ethical principles laid down in the Declaration of Helsinki.

12.2 Informed Consent Procedures

Eligible subjects may only be included in the study after providing written (witnessed, where required by law or regulation), IRB/IEC/REB-approved informed consent, or, if incapable of doing so, after such consent has been provided by a legally acceptable representative of the subject. Informed consent must be obtained before conducting any study-specific procedures (i.e. all of the procedures described in the protocol). The process of obtaining informed consent should be documented in the subject source documents.

Novartis Vaccines Institute for Global Health will provide to investigators a separate document with a proposed informed consent form that complies with the ICH GCP guideline and regulatory requirements and is considered appropriate for this study. Any changes to the proposed consent form suggested by the investigator must be agreed to by NVGH before submission to the IRB/IEC/REB, and a copy of the approved version must be provided to the NVGH monitor after IRB/IEC/REB approval.

Women of child bearing potential should be informed that taking the study vaccine may involve unknown risks to the fetus if pregnancy were to occur during the study and agree that in order to participate in the study they must adhere to the contraception requirements indicated in the protocol for the duration of the study. If case of doubts on the ability of a subject to adhere to these requirements, that subject should not be enrolled in the study.

12.3 Responsibilities of the Investigator and IRB/IEC/REB

The protocol and the proposed informed consent form must be reviewed and approved by a properly constituted Institutional Review Board/Independent Ethics Committee/Research Ethics Board (IRB/IEC/REB) before study start. A signed and dated statement that the protocol and informed consent have been approved by the IRB/IEC/REB must be given to NVGH before study initiation. Prior to study start, the investigator is required to sign a protocol signature page confirming his/her agreement to conduct the study in accordance with the protocol and all of the instructions and procedures referred to in this protocol and to give access to all relevant data and records to NVGH monitors, auditors, designated agents of NVGH, IRBs/IECs/REBs, and regulatory authorities as required. If an inspection of the clinical site is requested by a regulatory authority, the investigator must inform NVGH immediately that this request has been made.

12.4 Protocol Adherence

Investigators will apply due diligence to avoid protocol deviations. Under no circumstances should the investigator contact NVGH or its agents, if any, monitoring the trial to request approval of a protocol deviation, as no authorized deviations are permitted. If the investigator feels a change to the protocol would improve the conduct of the study this must be considered a protocol amendment, and unless such an amendment is agreed

upon by NVGH and approved by the IRB/IEC/REB it cannot be implemented. All significant protocol deviations will be recorded and reported in the CSR.

12.5 Protocol Amendments

An amendment is a written description of change(s) to or formal clarification of a study protocol which may impact the conduct of the clinical study, potential benefit of the clinical study, or may affect subject safety, including changes of study objectives, study design, subject population, sample sizes, study procedures, or significant administrative aspects. An administrative change of a study protocol is a minor correction or clarification that has no significant impact on the way the clinical study is to be conducted and no effect on subject safety (e.g., change of telephone number(s), logistical changes). Protocol amendments must be approved by NVGH, Health Authorities where required, and the IRB/IEC/REB. In cases when the amendment is required in order to protect subjects' safety, the amendment can be implemented prior to IRB/IEC/REB approval. Notwithstanding the need for formal approval of a protocol amendment, the investigator is expected to take any immediate action required for the safety of any subject included in this study, even if this action represents a deviation from the protocol. In such cases, NVGH should be notified of this action and the IRB/IEC/REB at the study site should be informed within 10 working days.

13.0 REFERENCE LIST

(1) Typhoid vaccines: WHO position paper. Weekly epidemiological record 6[83], 49-60. 8-2-2008.

(2) Keitel WA, Bond NL, Zahradnik JM, Cramton TA, Robbins JB. Clinical and serological responses following primary and booster immunization with Salmonella typhi Vi capsular polysaccharide vaccines. Vaccine 1994; 12(3):195-199.

(3) Dintzis RZ. Rational design of conjugate vaccines. Pediatr Res 1992; 32(4):376-385.

(4) Lin FY, Ho VA, Khiem HB, Trach DD, Bay PV, Thanh TC et al. The efficacy of a Salmonella typhi Vi conjugate vaccine in two-to-five-year-old children. N Engl J Med 2001; 344(17):1263-1269.

(5) Kanungo S, Dutta S, Sur D. Epidemiology of typhoid and paratyphoid fever in India. J Infect Dev Ctries 2008; 2(6):454-460.

(6) Zhang XL, Jeza VT, Pan Q. Salmonella typhi: from a human pathogen to a vaccine vector. Cell Mol Immunol 2008; 5(2):91-97.
