# Supplementary material for: Safety, Immunogenicity and Dose Ranging of a New Vi-CRM197 Conjugate Vaccine against Typhoid Fever: Randomized Clinical Testing in Healthy Adults
Source: PLoS One. 2011 Sep 30;6(9):e25398. doi: 10.1371/journal.pone.0025398 (PMC3184126; doi:10.1371/journal.pone.0025398)
Supplement: Protocol S2 — Trial Protocol of Phase 2 Trial (PDF) [file pone.0025398.s002.pdf]

CLINICAL STUDY PROTOCOL *H01_04TP*

Version 1.0 Dated 28th July 2010

EUDRACT No 2010-021874-12

BB-IND No *NA*

A Phase 2, Randomized, Observer–blind, Controlled, Single Center Study to Evaluate the Safety and Immunogenicity of Three Formulations of the NVGH Glycoconjugate Vaccine against S. Typhi in Adult Subjects 18 to 40 Years of Age.

Property of Novartis Vaccines Institute for Global Health

Confidential

May not be used, divulged, published or otherwise disclosed without written consent of Novartis Vaccines Institute for Global Health.

PROTOCOL SYNOPSIS [H01_04TP VERSION 1.0]

**Name of Sponsor** Novartis Vaccines Institute for Global Health	**Protocol number:** H01_04TP	**Eudract number:** 2010-021874-12	**Date of Protocol Synopsis:** 09 JUL 10
**Title of Study:** A Phase 2, Randomized, Observer–blind, Controlled, Single Center Study to Evaluate the Safety and Immunogenicity of Three Formulations of the NVGH Glycoconjugate Vaccine against S. Typhi in Adult Subjects 18 to 40 Years of Age.			
**Publication (reference):** None			
**Study Period:** Each subject will participate in the study for 28 days after vaccination.	**Clinical Phase:** Phase 2		

Rationale:

Despite the availability of S.Typhi vaccines for older children and adults, typhoid fever remains a major health problem in developing countries. The World Health Organization (WHO) estimated the global typhoid disease burden at 21 million cases annually with 200 to 600 thousand deaths per year[1]. In many areas of Asia, Africa and Latin America, high incidence rates for S.Typhi have been reported and this disease remains a major public health problem. Of those infected, children of school age or younger are disproportionately affected. Serious complications occur in up to 10% of the cases, resulting in death in 1% to 4% of younger children.

The currently licensed vaccines against S.Typhi are the oral live attenuated and the Vi polysaccharide vaccines. As purified Vi polysaccharide is a T independent antigen, the currently licensed vaccines are not able to prime vaccinees for immunological memory, can not be boosted by repeated vaccination [2] and are poorly immunogenic in the very young children; therefore they are only recommended for children≥ 2 years of age. The coupling of polysaccharide antigens to carrier proteins transforms the antigen, so called conjugate polysaccharide, into a T-cell dependent antigen, capable of inducing an immunological memory and an adequate immune response in infants and young children[3].

NVGH Vi-CRM_{197} is a glycoconjugated vaccine, based on chemical conjugation of the Vi polysaccharide with the CRM_{197} carrier protein. Due to the T-cell-dependent immunological properties of glycoconjugates, this vaccine should be able to overcome the limitations of the currently available polysaccharide vaccines and offer an effective tool for immunization of not only young children but also infants and toddlers less than 2 years of age, a longer-lasting immune response and a boostable memory response against S. Typhi. Consequently the NVGH Vi-CRM conjugate vaccine may be

**Name of Sponsor** Novartis Vaccines Institute for Global Health	**Protocol number:** H01_04TP	**Eudract number:** 2010-021874-12	**Date of Protocol Synopsis:** 09 JUL 10

integrated into EPI routine infant vaccination.

The NVGH candidate Vi-CRM_{197} vaccine results from the conjugation of two well characterized components: the Vi polysaccharide (which is a widely licensed vaccine) and the mutant of the diphtheria toxin protein (CRM_{197}), a carrier protein used in many licensed vaccines, including the *H. influenzae* type b, *N. meningitidis* and *S. pneumoniae* vaccines. The NVGH Vi antigen is obtained from a strain of *Citrobacter* -------------, unlike the licensed Vi polysaccharide vaccine which is derived from a Salmonella Typhi strain. The Vi molecules obtained from the two strains are chemically undistinguishable.

A Phase 1 trial, H01_01TP, is ongoing to evaluate the safety and immunogenicity profiles of a new Vi-CRM_{197} conjugate vaccine in healthy human adults in comparison with the currently licensed Vi polysaccharide vaccine (Typherix, GSK, Belgium). The preliminary safety and immunogenicity data from this trial have revealed that, compared to the licensed polysaccharide vaccine, the conjugate vaccine is significantly more immunogenic, but is also associated with a higher local reactogenicity. The analysis of the safety and immunogenicity results suggests that a lower antigen concentration of Vi-CRM_{197} should be selected for further development.

The present Phase 2 trial is aimed to evaluate, in healthy adults, the safety and the immunogenicity of three different vaccine formulations containing lower concentrations of Vi-CRM_{197} compared to that used in the Phase 1 trial. The Vi polysaccharide vaccine (Typherix, GSK, Belgium) is used as licensed control.

Study Agent/Intervention Description: NVGH *S. Typhi* glyconjugate vaccine (Vi-CRM_{197}):

The test vaccine Vi-CRM_{197} is available in single dose vials containing 0.7 ml of injectable solution.

A 0.5 ml dose of Vi- CRM_{197} vaccine contains 25 μg of Vi polysaccharide conjugated to approximately 25 μg of CRM_{197}. The vaccine does not contain any adjuvant, stabilizer or preservative.

In the present trial, the vaccine will be used at three different antigen concentrations (Vaccine Dose A, Vaccine Dose B and Vaccine Dose C) which will be obtained by bed side mixing of **Vi-CRM_{197} - 25 μg.**

Objectives:

**Name of Sponsor** Novartis Vaccines Institute for Global Health	**Protocol number:** H01_04TP	**Eudract number:** 2010-021874-12	**Date of Protocol Synopsis:** 09 JUL 10

Safety Objectives:

To evaluate the safety profile of three different dose levels of Vi-CRM_{197} in adults compared to that of the licensed Vi polysaccharide vaccine (Typherix, GSK), by measuring rates of post immunization reactions and adverse events.

Immunogenicity Objectives:

To evaluate the immunogenicity profile of three different dose levels of Vi-CRM_{197} in adults compared to that of the licensed Vi polysaccharide vaccine (Typherix, GSK), by measuring enzyme-linked immunosorbent assay (ELISA), at 28 days post-immunization.

Methodology:

This will be a randomized, observer blind, active vaccine-controlled, single center, phase 2 clinical trial.

Only subjects giving informed consent with negative screening tests for drug addiction and women with negative pregnancy test will be randomized.

Enrolled subjects will be randomized at a 1:1:1:1 ratio to receive, at day 1 a single intramuscular injection of either one of the three formulations of the investigational NVGH Vi-CRM_{197} conjugate vaccine (groups A, B, C) or the licensed Vi polysaccharide vaccine (group D), using a syringe with a 25 mm needle. Each randomized subject will have 10 ml of blood drawn for serology at day 1 following randomization, and will be vaccinated with the assigned vaccine after the blood draw. Another 10 ml of blood will be drawn for serology at day 28 post immunization (see Appendix I).

Reactogenicity will be assessed daily during the first week following vaccination: local and systemic reactions occurring at day 1 to day 7 will be collected and recorded in the diary card. On day 8, all information on local or systemic reactions and adverse events (AEs) recorded in the diary cards will be collected on an ad-hoc form via a structured telephone call. The ad-hoc form will be kept as a source documentation for reconciliation with information in diary card brought in by the subject at visit 2 (day 28).
The overall safety follow-up will be 4 weeks (28 days) for each subject: all adverse events (AE) will be collected and documented for the entire duration of the study (28 days).

Summary of Study Vaccination and Blood Sampling

Name of Sponsor	Protocol number:	Eudract number:	Date of Protocol Synopsis:
Novartis Vaccines Institute for Global Health	H01_04TP	2010-021874-12	09 JUL 10

Groups	*Vaccine*	*Number of subjects*	*Day 1*	*Day 28*
Group A	*NVGH Vi-CRM$_{197}$ Dose A*	*22*	*Blood sampling Vaccination*	*Blood sampling*
Group B	*NVGH Vi-CRM$_{197}$ Dose B*	*22*	*Blood sampling Vaccination*	*Blood sampling*
Group C	*NVGH Vi-CRM$_{197}$ Dose C*	*22*	*Blood sampling Vaccination*	*Blood sampling*
Group D	*Licensed Vi polysaccharide vaccine*	*22*	*Blood sampling Vaccination*	*Blood sampling*

Number of Subjects planned:

A total of 88 subjects (22 for each of the four groups) are planned for enrolment into the study. Assuming a 10% drop out rate, there will be approximately 80 evaluable subjects (20 for each of the four study groups). Subjects withdrawn or lost to follow up will not be replaced.

Subject Population:

The study population will consist of male and female adult subjects aged 18 to 40 years. Female subjects must use birth control during study participation and one month before study start. Those with immunosuppressive conditions including the use of chronic high dose inhaled steroids will not be included.

Subject Characteristics and Main Criteria for Inclusion and Exclusion:

Inclusion Criteria

1. Males and females of age ≥18 to ≤40 years.
2. Individuals who, after the nature of the study have been explained to them, have given written consent according to local regulatory requirements.
3. Individuals in good health as determined by the outcome of medical history, physical examination and clinical judgment of the investigator.
4. Individuals with negative urine screening tests for drug addition as follows:

Opiate	Negative
Cocaine	Negative
Amph/Metamphetamine	Negative
Cannabinoides	Negative

5. If women, use of birth control one month before study start, a negative pregnancy

**Name of Sponsor** Novartis Vaccines Institute for Global Health	**Protocol number:** H01_04TP	**Eudract number:** 2010-021874-12	**Date of Protocol Synopsis:** 09 JUL 10

test and willingness to use birth control measures for the entire study duration.

Exclusion Criteria

1. Individuals with behavioral or cognitive impairment or psychiatric disease that, in the opinion of the investigator, may interfere with the subject's ability to participate in the study.
2. Individuals with any progressive or severe neurological disorder, seizure disorder or Guillain-Barré syndrome.
3. Individuals who are not able to understand and to follow all required study procedures for the whole period of the study.
4. Individuals with history of any illness that, in the opinion of the investigator, might interfere with the results of the study or pose additional risk to the subjects due to participation in the study.
5. Individuals with known or suspected HIV infection or HIV related disease, with history of an autoimmune disorder or any other known or suspected impairment /alteration of the immune system, or under immunosuppressive therapy including use of systemic corticosteroids or chronic use of inhaled high-potency corticosteroids within the previous 30 days, or were in chemotherapy treatment within the past 6 months.
6. Individuals with a known bleeding diathesis, or any condition that may be associated with a prolonged bleeding time.
7. Individuals with any serious chronic or progressive disease according to judgment of the investigator (e.g., neoplasm, insulin dependent diabetes, cardiac, renal or hepatic disease).
8. Individuals who have any malignancy or lymphoproliferative disorder.
9. Individuals with history of allergy to vaccine components.
10. Individuals participating in any clinical trial with another investigational product 30 days prior to first study visit or intent to participate in another clinical study at any time during the conduct of this study.
11. Individuals who have previously received any vaccines against typhoid fever (either oral live attenuated or injectable vaccines)
12. Individuals who received any other vaccines within 4 weeks prior to enrolment in this study or who are planning to receive any vaccine within 4 weeks from the study vaccine

**Name of Sponsor** Novartis Vaccines Institute for Global Health	**Protocol number:** H01_04TP	**Eudract number:** 2010-021874-12	**Date of Protocol Synopsis:** 09 JUL 10

13. Individuals who have received blood, blood products and/or plasma derivatives including parenteral immunoglobulin preparations in the past 12 weeks.
14. Individuals who are part of study personnel or close family members to the personnel conducting this study.
15. Individuals with body temperature $\geq$ 38.0 degrees Celsius within 3 days of intended study immunization.
16. BMI > 35 $kg/m^{2.}$
17. Individuals with history of substance or alcohol abuse within the past 2 years.
18. Women who are pregnant or breast-feeding or of childbearing age who have not used any birth control measure one month prior to study start or do not plan to use acceptable birth control measures, for the duration of the study.
19. Females with history of stillbirth, neonatal loss, or previous infant with anomaly.
20. Individuals who have a previously ascertained or suspected disease caused by S. Typhi.
21. Individuals who have had household contact with/and or intimate exposure to an individual with laboratory confirmed S. Typhi.
22. Any condition which, in the opinion of the investigator may interfere with the evaluation of the study objectives.

Vaccines:

Test Vaccine

The test vaccine is NVGH S. Typhi glyconjugate vaccine (Vi-CRM_{197}). The vaccine is available in single dose vials containing 0.7 ml of injectable solution. Each 0.5 ml immunization dose of Vi-CRM_{197} contains 25 µg of Vi polysaccharide conjugated to CRM_{197} (**Vi-CRM_{197} - 25 µg**) The vaccine does not contain any adjuvant, stabilizer or preservative.

In the present trial, the vaccine will be used at three different antigen concentrations (Vaccine Dose A, Vaccine Dose B and Vaccine Dose C) which will be obtained by bed side mixing of **Vi-CRM_{197} - 25 µg.**

Vi-CRM_{197} – Dose A

Each 0.5 ml dose of **Vi-CRM_{197} – Dose A** contains **12.5 µg** of Vi polysaccharide

**Name of Sponsor** Novartis Vaccines Institute for Global Health	**Protocol number:** H01_04TP	**Eudract number:** 2010-021874-12	**Date of Protocol Synopsis:** 09 JUL 10

conjugated to CRM_{197}. The vaccine does not contain any adjuvant, stabilizer or preservative.

Vi-CRM_{197} – Dose A is obtained by mixing 0.5 ml of Vi-CRM_{197} - 25 μg with 0.5 ml of saline solution (resulting in 1.0 ml of **Vi-CRM_{197} – Dose A).**

The subjects randomized to receive Vi-CRM_{197} – Dose A will receive one 0.5 ml (i.e., 12.5 μg of Vi-CRM_{197}) IM injection in the deltoid of the non-dominant arm (Group A).

Vi-CRM_{197} – Dose B

Each 0.5 ml dose of **Vi-CRM_{197} – Dose B** contains **5.0 μg** of Vi polysaccharide conjugated to CRM_{197}. The vaccine does not contain any adjuvant, stabilizer or preservative.

Vi-CRM_{197} – Dose B is obtained by mixing 0.5 ml of Vi-CRM_{197} - 25 μg with 2.0 ml of saline solution (resulting in 2.5 ml of **Vi-CRM_{197} – Dose B**).

The subjects randomized to receive Vi-CRM_{197} – Dose B will receive one 0.5 ml (i.e., 5.0 μg of Vi-CRM_{197}) IM injection in the deltoid of the non-dominant arm (Group B).

Vi-CRM_{197} – Dose C

Each 0.5 ml dose of **Vi-CRM_{197} – Dose C** contains **1.25 μg** of Vi polysaccharide conjugated to CRM_{197}. The vaccine does not contain any adjuvant, stabilizer or preservative.

Vi-CRM_{197} – Dose C is obtained by mixing 0.5 ml of Vi-CRM_{197} - 25 μg with 9.5 ml of saline solution (resulting in 10.0 ml of **Vi-CRM_{197} – Dose C**).

The subjects randomized to receive Vi-CRM_{197} – Dose C will receive one 0.5 ml (i.e., 1.25 μg of Vi-CRM_{197}) IM injection in the deltoid of the non-dominant arm (Group C).

<u>Reference Vaccine</u>

Reference vaccine is the Vi polysaccharide vaccine (Typherix®, GlaxoSmithKline). A 0.5 ml dose of Typherix® contains 25 μg of Vi.

One 0.5 ml dose out of single dose pre-filled syringes of Vi vaccine will be injected IM in the deltoid of the non-dominant arm (Group D).

<u>Concomitant Vaccines or Treatment</u>

No concomitant vaccines or treatment will be used in this study.

**Name of Sponsor**<br>Novartis Vaccines Institute for Global Health	**Protocol number:**<br>H01_04TP	**Eudract number:**<br>2010-021874-12	**Date of Protocol Synopsis:**<br>09 JUL 10

Immunogenicity Endpoints

The measures of immunogenicity, against the Vi antigen of S. Typhi, will include:

- Geometric mean concentrations (GMCs), pre- and post-vaccination, as determined by ELISA, and applicable geometric mean ratios between post- and pre-vaccination samples.

- Seroconversion rate: percentage of subjects achieving at least a four-fold rise in ELISA antibody concentration in the post-vaccination blood sample

Safety Endpoints (criteria for assessing safety endpoints)

The measures of safety will include:

- Numbers and percentage of subjects with solicited local and systemic adverse reactions as well as numbers and percentage of subjects with reported unsolicited adverse events and serious adverse events.

Solicited local reactions include erythema, induration and pain at injection site; solicited systemic reactions include headache, arthralgia, chills, fatigue, malaise, myalgia, and fever as measured by axillary temperature for Day 1 through 7 of the study.

All local and systemic reactions will be collected for 7 days after immunization (i.e., days 1 to 7). All AEs will be collected throughout the study duration (28 days).

Interim Analysis:

No interim analysis will be performed.

Data Monitoring Committee:

A data safety monitoring board (DSMB) will not be utilized for this study.

APPENDIX I - TIMES AND EVENTS TABLE

Study Periods	Enrolment & Vaccination	Post-Vaccination
**Visit No.**	**1**	**2**
**Clinic Visit? (Yes/No)[a]**	Yes	Yes
**Study Day**	1	28
**Study Visit Window**	NA	+7
ICF	X	
Exclusion/Inclusion	x[b]	
Medical history	x[b]	
Physical examination[c]	x[b]	X
Randomization	x[b]	
**Vaccine administered**	X	
Serology Blood draw [max 10 ml]	x[b]	X
Urinalysis for drug addition	x[b]	X
Pregnancy test	x[b]	X
Diary Card Dispensed	X	
Diary Card Collected and/or Reviewed		X
Assess Local/ Systemic Reactions[d, e]	X	
Assess AEs and SAEs [f]	X	X
Concomitant medications[g]	X	X
Study termination[h]		X

a. Clinic visit "NO" refers to telephone contact only with subject
b. Performed prior to vaccination
c. Physical examination (including injection site or intended injection site) must be performed by a qualified health professional designated within the Site Responsibility Delegation Log. Complete physical examination will be performed at Day 1 and Day 28
d. Data on local and systemic reactions will be collected by the study personnel for all subjects at 1 hour post-injection. Subjects will record local and systemic reactions on the diary card daily for 7 days after study vaccination
e. On Day 8, collect all information on local or systemic reactions and adverse events (AEs) recorded in diary card on an ad-hoc form via a structured telephone call. The ad-hoc form will be kept as a source documentation for reconciliation with information in diary card brought in by the subject at Visit 2.
f. Assess AEs and SAEs according to Safety Assessment Table (Table 6.2.1)
g. Collect concomitant medications/vaccines according to Safety Assessment Table (Table 6.2.1)
h. Any subject who terminates the study earlier is recommended to undergo study-termination procedures.

TABLE OF CONTENTS

PROTOCOL SYNOPSIS [H01_04TP VERSION 1.0] 2
APPENDIX I - TIMES AND EVENTS TABLE 10
LIST OF ABBREVIATIONS AND DEFINITIONS OF TERMS 14
1.0 BACKGROUND AND RATIONALE 15
2.0 OBJECTIVES 16
2.1 Safety Objectives 16
2.2 Immunogenicity Objectives 16
3.0 STUDY DESIGN AND INVESTIGATIONAL PLAN 16
3.1 Overview of Study Design 16
3.2 Discussion of Overall Study Design 17
3.2.1 Study sites 17
3.2.2 Target Population 17
3.2.3 Recruitment 18
3.3 Study Procedures and Flowchart 18
3.3.1 Subject Numbering 22
3.3.2 Method of Assignment to Study Groups 22
3.3.3 Blinding procedures 22
3.3.4 Vaccine Supply, Storage, Tracking and Labeling 23
3.3.5 Processing, Labeling and Storage of Serum Samples for Serology 24
3.4 Duration of Subject's Expected Participation in the Entire Study 24
3.5 Stopping/Pausing Rules 24
4.0 SELECTION OF STUDY POPULATION 24
4.1 Inclusion Criteria 24
4.2 Exclusion Criteria 25
4.3 Withdrawal of Subjects from Therapy or Assessment 26
5.0 TREATMENT OF SUBJECTS 27
5.1 Investigational Vaccine(s) 27
5.1 Investigational Vaccine - NVGH S. Typhi conjugate vaccine 27
5.2 Control Vaccine – GSK Typherix® 28

5.3 Concomitant Vaccines or Treatment 28
5.4 Vaccines Preparation and Administration 29
5.5 Other Concomitant Treatment or Vaccines 30
5.6 Vaccination Compliance 30
6.0 EFFICACY/IMMUNOGENICITY AND SAFETY ASSESSMENTS 31
6.1 Appropriateness of Measurements 31
6.1.1 Immunogenicity 31
6.1.2 Methods, Criteria and Timing for Assessing and Recording Immunogenicity Parameters 31
6.2 Safety Parameters 31
6.2.1 Local and Systemic Reactions 33
6.2.2 Adverse Events 33
6.2.3 Serious Adverse Events 34
6.2.4 Subject’s Diary Card 35
6.2.6 Methods and Timing for Assessing and Recording Safety Parameters 35
6.3 Data Monitoring Committee 37
7.0 STATISTICAL PLAN 37
7.1 Statistical Hypothesis 37
7.2 Sample Size and Power Considerations 37
7.3 Population for Analysis 39
7.4 Analysis of Demographic and Baseline Characteristics 40
7.5 Analysis of Efficacy/Immunogenicity Endpoints 40
7.5.1 Description of Response Variables 40
7.5.2 Statistical Methods for Efficacy/Immunogenicity Variables 40
7.6 Analysis of Safety (Endpoints) and Tolerability 41
7.6.1 Analysis of Extent of Exposure 41
7.6.2 Analysis of Local and Systemic Reactions 41
7.6.3 Analysis of Other Adverse Events 42
7.7 Planned Interim Analysis 42
8.0 STUDY MONITORING, AUDITING AND DOCUMENTATION 42
8.1 Study Monitoring 43

8.2 Source Data Verification 43
9.0 DATA MANAGEMENT..44
9.1 Data Handling Procedures 44
9.2 Documentation of Study Findings 44
9.3 Data Protection 45
10.0 RECORD RETENTION..45
11.0 USE OF INFORMATION AND PUBLICATION 46
12.0 ETHICS...46
13.0 REFERENCE LIST 48

LIST OF ABBREVIATIONS AND DEFINITIONS OF TERMS

Ab	Antibody
AE	Adverse Event
BCDM	Biostatistics and Clinical Data Management
BMI	Body Mass Index
CD	Compact Disc
CHMP	Committee for Medicinal Products for Human Use
CRA	Clinical Research Associate
CRF	Case Report Form
CRM	Cross-Reacting Material
DCF	Data Clarification Form
DSMB	Data Safety Monitoring Board
EC	Ethics Committee
eDC	electronic Data Capture
EDT	Electronic Data Transfer
ELISA	Enzyme-Linked Immunosorbent Assay
EPI	Expanded Program on Immunization
GCP	Good Clinical Practice
GMC	Geometric Mean Concentration
GMP	Good Manufacturing Practice
HEE	Hidden Entry Envelopes
ICD	International Classification of Diseases
ICF	Informed Consent Form
ICH	International Conference on Harmonization
IEC	Independent Ethics Committee
IM	Intramuscular
IND	Investigational New Drug
IRB	Institutional Review Board
IUD	Intrauterine Device
MD	Medical Doctor
MedDRA	Medical Dictionary for Regulatory Activities
mITT	Modified Intention-To-Treat
NVGH	Novartis Vaccines Institute for Global Health
PP	Per Protocol
REB	Regional Ethics Board
SAE	Serious Adverse Event
SOP	Standard Operating Procedure
SUSAR	Suspected Unexpected Serious Adverse Reaction
WHO	World Health Organization

1.0 BACKGROUND AND RATIONALE

Salmonella is a rod-shaped gram-negative facultative anaerobe bacterium belonging to the *Enterobacteriaceae* family. *Salmonella enterica* serovar Typhi (S. Typhi) is pathogenic exclusively for humans and cause systemic infections and typhoid fever, a severe, contagious and life threatening systemic disease which may result in persistent fever with or without severe complications [5;6].

Despite the availability of S. Typhi vaccines for older children and adults, typhoid fever remains a major health problem in developing countries. The World Health Organization (WHO) estimated the global typhoid disease burden at 21 million cases annually with 200 to 600 thousand deaths per year [1]. In many areas of Asia, Africa and Latin America, high incidence rates for S.Typhi have been reported and this disease remains a major public health problem. Of those infected, children of school age or younger are disproportionately affected. Serious complications occur in up to 10% of the cases, resulting in death in 1% to 4% of younger children.

The existence of multidrug-resistant S. Typhi, that was found to be resistant to treatment with most of commonly used antibiotics such as chloramphenicol, ampicillin, streptomycin and tetracycline, is a serious and growing problem in the treatment of typhoid, especially in the developing country, where the vaccination has been proven to be an effective way of controlling typhoid, especially in vulnerable group [5].

The currently licensed vaccines against S. Typhi are the oral live attenuated and the Vi polysaccharide vaccines. As purified Vi polysaccharide is a T independent antigen, the currently licensed vaccines are not able to prime vaccines for immunological memory, can not be boosted by repeated vaccinations [2] and are poorly immunogenic in the very young children; therefore they are only recommended for children ≥2 years of age. The coupling of polysaccharide antigens to carrier proteins transforms the antigen, so called conjugate polysaccharide, into a T-cell dependent antigen, capable of inducing an immunological memory and an adequate immune response in infants and young children[3].

NVGH Vi-CRM_{197} is a glycoconjugated vaccine, based on chemical conjugation of the Vi polysaccharide with the CRM_{197} carrier protein. Due to the T-cell-dependent immunological properties of glycoconjugates, this vaccine should be able to overcome the limitations of the currently available polysaccharide vaccines and offer an effective tool for immunization of not only young children but also infants and toddlers less than 2 years of age, a longer-lasting immune response and a boostable memory response against S. typhi. Consequently the NVGH Vi-CRM conjugate vaccine may be integrated into EPI routine infant vaccination.

The NVGH candidate Vi-CRM_{197} vaccine results from the conjugation of two well characterized components: the Vi polysaccharide (which is a widely licensed vaccine) and the mutant of the diphtheria toxin protein (CRM_{197}), a carrier protein used in many

licensed vaccines, including the *H. influenzae* type b, *N. meningitidis* and *S. pneumoniae* vaccines. The NVGH Vi antigen is obtained from a strain of *Citrobacter* ------------, unlike the licensed Vi polysaccharide vaccine which is derived from a *Salmonella* Typhi strain. The Vi molecules obtained from the two strains are chemically undistinguishable.

A Phase 1 trial, H01_01TP, is ongoing to evaluate the safety and immunogenicity profiles of a new Vi-CRM_{197} conjugate vaccine in healthy human adults in comparison with the currently licensed Vi polysaccharide vaccine (Typherix, GSK, Belgium). The preliminary safety and immunogenicity data from this trial have revealed that, compared to the licensed polysaccharide vaccine, the conjugate vaccine is significantly more immunogenic, but is also associated with a higher local reactogenicity. The analysis of the safety and immunogenicity results suggests that a lower antigen concentration of Vi-CRM_{197} should be selected for further development.

The present Phase 2 trial is aimed to evaluate, in healthy adults, the safety and the immunogenicity of three different vaccine formulations containing lower concentrations of Vi-CRM_{197} compared to that used in the Phase 1 trial. The Vi polysaccharide vaccine (Typherix, GSK, Belgium) is used as licensed control.

The trial will be conducted in compliance with the protocol, GCP and the applicable regulatory requirements.

2.0 OBJECTIVES

2.1 Safety Objectives

To evaluate the safety profile of three different dose levels of Vi-CRM_{197} in adults compared to that of the licensed Vi polysaccharide vaccine (Typherix, GSK), by measuring rates of post immunization reactions and adverse events.

2.2 Immunogenicity Objectives

To evaluate the immunogenicity profile of three different dose levels of Vi-CRM_{197} in adults compared to that of the licensed Vi polysaccharide vaccine (Typherix, GSK), by measuring enzyme-linked immunosorbent assay (ELISA), at 28 days post-immunization.

3.0 STUDY DESIGN AND INVESTIGATIONAL PLAN

3.1 Overview of Study Design

This is a phase 2, randomized, observer–blind, active vaccine controlled study to evaluate the safety and immunogenicity of three formulations of the NVGH Glycoconjugate Vaccine against S. Typhi in male and female subjects 18 to 40 years of age.

The study has two visits:

- **Enrolment and Vaccination:** Subjects, who provide a written informed consent, will be screened for eligibility, enrolled and randomized to receive one of the four vaccines on Visit 1.

- **Post Vaccination follow up:** Subjects will be followed for 28 (+7) days after vaccination.

3.2 Discussion of Overall Study Design

This phase 2 study utilizes several standard features of clinical study design intended to reduce bias, including the random assignment of subjects to treatment groups.

The active comparator, the Vi polysaccharide vaccine, was selected to enable comparison of the study treatment to a similar licensed polysaccharide vaccine, since there is no other S.Typhi conjugate vaccine currently available. There are no placebo groups in this study since there would be no immunogenic response and no local or systemic reactions, which would enable a comparison with the properties of the Vi-CRM vaccine.

The frequency of study visits and assessments are consistent with previous studies investigating the tolerability and immunogenicity of conjugate vaccines. In those studies, the majority of local and systemic reactions occurred within 2 days of vaccination and lasted no more than 4 days and therefore any local or systemic reactions in this study should be captured at Visit 2.

3.2.1 Study sites

The study will be performed at one study site.

Administrative part of the study will be performed at:
Centre for the Evaluation of Vaccination

The study will be performed at:

Research Unit of the University Hospital
Campus 3 Eiken Universiteitsplein, 1 (loc. R2.14), B-2610 Antwerpen
www.ua.ac.be/cev
www.vhpb.org

3.2.2 Target Population

The study will be performed in adults 18-40 years of age. A total of 88 subjects (22 subjects per group) are planned for enrolment into the study. Assuming a 10% drop-out rate, there will be approximately 80 evaluable subjects (20 subjects per group).

3.2.3 Recruitment

The Centre for the Evaluation of Vaccination existing database of study volunteers will be used. Further recruitment will be performed through intranet system of the University of Antwerp and the University Hospital of Antwerp.

Posters will be posted in buildings of the University of Antwerp as well as the University Hospital. Advertisements on local Antwerp TV channel will be also shown.

3.3 Study Procedures and Flowchart

The study is divided into two visits: Enrolment and Vaccination & Post Vaccination follow up (28 Days).

Enrolment and Vaccination

Visit 1 (Day 1):

The following procedures will be performed:

- Study procedures should be explained and informed consent must be obtained from the subject prior to any study related procedure.
- Record medical history of the subject. The medical history should include details of any significant present and past diagnosis in the last 12 months (for details refer to table 6.2.1).

  Perform complete physical examination of the subject including but not limited to: blood pressure, lungs and heart. The physical examination must be performed by a qualified health professional designated within the Site Responsibility Delegation Log.
  Perform assessment of subject's axillary temperature.
  Note: If axillary temperature is ≥38.0 °C just prior to vaccination when measured using a validated device, vaccination must be postponed until the fever has resolved.

- Record all concomitant medications and vaccines (received within last 4 weeks), for details refer to table 6.2.1.
- Confirm and record use of birth control measure one month prior to study start.
- Perform a Drug Addiction Screening test
- Perform urine pregnancy test and confirm that the female subject is not pregnant.

- Indicate that subjects of child-bearing potential must practice appropriate birth control from this visit until study conclusion.
  - Female of childbearing potential is defined as a pre-menopausal female capable of becoming pregnant. This does not include females who meet any of the following conditions: (1) menopause at least 2 years earlier, (2) tubal ligation at least 1 year earlier, or (3) total hysterectomy.
  - Reliable birth control method is defined as hormonal (e.g., oral, injection, transdermal patch, implant, cervical ring), barrier (e.g., condom with spermicide or diaphragm with spermicide), intrauterine device (e.g., IUD), or monogamous relationship with partner who has been vasectomized for 6 months or more prior to the subject's study entry.
  - Sexual abstinence for the duration of the study (28 days).

- Confirm that the subject meets ALL inclusion and NO exclusion criteria.

- **Randomize** the subject by assigning the subject number.

- Collect 10 ml blood for serology before vaccine administration.

- Administer the vaccine according to the study group assigned by randomization. The injection will be administered by an unblinded study staff.

- During the 1 hour following injection, observe the subject for any immediate reaction to the vaccine. At the end of the observation period, assess for local/ systemic reactions.

- Dispense the diary card to the subject and explain in detail instructions for completion. Instruct the subject in how to record axillary temperature, report local and systemic reactions, AEs (Adverse Events), concomitant medications (prescription and non prescription). Also instruct the subject to notify the site immediately if they experience any SAEs (Serious Adverse Events) or event of concern to them.

- Confirm birth control measures and remind that subjects of child bearing potential must practice appropriate birth control from this visit until study conclusion.

- Schedule the subject for Visit 2.

Day 8 Safety Phone Call

- Collect all information on local or systemic reactions and adverse events (AEs) recorded in the diary card on an ad-hoc form via a structured telephone call. The ad-hoc form will be kept as a source documentation for reconciliation with information in diary card brought in by the subject at Visit 2.

Post Vaccination follow up

Visit 2: Day 28 (window + 7)

- Assess any AEs or SAEs since last visit.
- Perform the physical examination directed by medical history. The physical examination must be performed by a qualified health professional designated within the Site Responsibility Delegation Log.
- Record all concomitant medications administered as treatment of AE and all vaccines – excluding study vaccines (received since last visit).

  If the subject received concomitant medications not allowed in the study and/or vaccines, the protocol violation should be reported
- Collect subject's Diary Card. Review Diary Card with the subject for completeness, especially regarding descriptions of any local or systemic reactions AEs or concomitant medication use (reconciling with the ad-hoc form completed on Day 8). Record information obtained on appropriate source documents and CRF pages.
- Perform Urine Pregnancy test
- Collect 10 ml blood for serology.
- Inform the subject of study termination and complete Study Termination CRF.

Visit 1 (Enrolment & Vaccination) **Visit 2 (Post Vaccination follow up)**

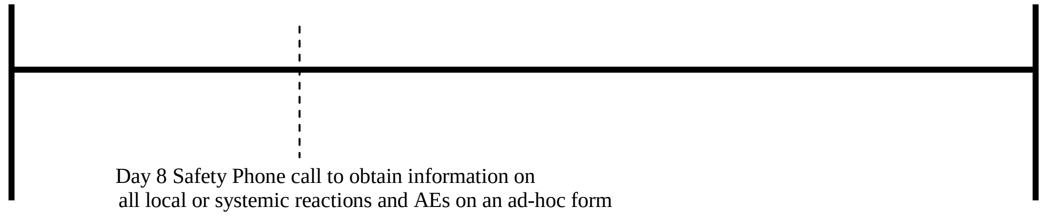

- ICF
- Medical History
- Previous and Concomitant Medications
- Baseline Physical Exam
- Drug Addiction Screening test
- Urine Pregnancy Test
- Inclusion/ Exclusion
- Blood draw (Serology)
- Randomization
- Vaccination
- Diary Card (Dispensed)
- Assess Immediate Local/Systemic Reactions

. Diary Card Collection and Review

. Follow up Physical Assessment
. Blood Draw (Serology)
. Assess AEs/ SAEs
. Concomitant Medications
. Urine Pregnancy Test
. Study Termination

3.3.1 Subject Numbering

Subjects who meet all inclusion criteria and none of the exclusion criteria will be randomized and given a **subject number** on Visit 1. A randomized subject will be identified by a 5-digit subject number. The first 2 digits will be 10 (corresponding to the investigator site number) and the last 3 digits identifying the subject within the site. The digits identifying the subject within the site are assigned sequentially, with 001 corresponding to the first subject enrolled at study site.

Once assigned to a subject, the subject number cannot be reused.

The investigator must record the names of the randomized subjects and their identifying numbers in a Subject Identification Code List.

To each subject enrolled in the study will be assigned a **subject code**. To respect data protection, the use of the initials will be avoided. Therefore, instead of using the subject's initials, the second letter of the first name followed by the second letter of the middle name or a dash (according to the convention adopted by the site) and then by the second letter of the last name (surname) will be used. If a subject does not have a middle name a dash should be included.

3.3.2 Method of Assignment to Study Groups

The eligible subjects will be randomized to the four study groups on a 1:1:1:1 ratio (see section 5.0 for vaccines composition).

Hidden Entry Envelopes (HEEs) containing vaccines group information will be used in this trial, thus preventing tampering and reading of the assigned group before the subject number is given.

Laboratories performing serology analysis will be blinded to individual participant group assignments.

3.3.3 Blinding procedures

This is an observer blind study: the subjects, investigator, and or other site personnel and sponsor staff are not informed of treatment group assignment, but there will be one unblinded site personnel who will have access to the vaccine assignment of subjects once they have been randomized.

The HEEs will be produced by the BCDM department using a system that automates the random assignment of treatment arms to subject numbers with a 1:1:1:1 allocation ratio. Designated unblinded personnel at the BCDM department will be responsible to the production of the randomization list and the HEEs. The HEEs will be delivered to the study site prior to the initiation of the study.

HEE will allow revealing the vaccination group assignment on a per-subject basis; they must be stored in a secure place and opened one by one, only after the subject has been found eligible to be randomized.

3.3.4 Vaccine Supply, Storage, Tracking and Labeling

Novartis Vaccines Institute for Global Health will supply the vaccines to the investigational site. Temperature will be monitored during the shipment. The investigator should acknowledge receipt of the study vaccines. Upon receipt, investigator or designee should ensure study vaccines are received in good condition. The investigator shall inform immediately the sponsors of any shipment temperature out of range.

The vaccines at the site must not be used before the appropriate shipping conditions have been checked and confirmed. Study vaccine will be labeled and will comply with the legal requirements of Belgium and international guidelines. Study vaccines must be handled properly and stored in a secure location to which only the investigator or designee have access.

All study vaccines must be stored in a safe, locked, and secure place with no access by unauthorized personnel. They must be kept in the refrigerator (+2°C to +8°C) and **must not be frozen**. Storage temperature should be monitored every day. Access to a back-up refrigerator in case of power failure/breakdown is necessary.

Vaccines that have been stored differently from the sponsor’s recommendations **must not** be used unless the sponsor provides written authorization for use. In the event that the use cannot be authorized, vaccine supply must be replaced with fresh stock supplied by the sponsor.

The investigator should ensure that the vaccines delivered to the site are used only in accordance with the approved protocol. Monitoring of vaccine accountability will be performed by the unblinded study monitor during site visits.

The investigator should maintain an accurate record of products delivery to the site, the inventory at the site, the administration to the subjects, and the return to the sponsor, or destruction, of study vaccines. At the conclusion, and as appropriate during the course of the study, the investigator will return to the sponsor, or destroy at study site (as per sponsor requirements and SOP) all used and unused study vaccines, packaging and supplementary labels. If the unused study vaccines are disposed at the site, the investigator should provide a copy of the site’s procedure for destruction of hazardous material and documentation of the destruction.

Study vaccines will be labeled to comply with the legal requirements of Belgium and international guidelines. All study vaccines must be stored according to the instructions specified on the labels.

3.3.5 Processing, Labeling and Storage of Serum Samples for Serology

For measuring anti-Vi antibody concentration (ELISA) in the serum, 10 ml of blood will collected from each subject at visits 1 (pre vaccination) and 2 (28 days post vaccination). Additional serology tests to evaluate the immunogenicity of the study vaccines may be performed if deemed necessary by the sponsor.

Blood samples must be collected in the appropriate manner, using exclusively materials and guidelines supplied by the sponsor. The investigator must ensure that his/her personnel and the laboratory(ies) under his/her supervision comply with this requirement.

Serum samples will be stored frozen at -20ºC. Shipment to the different laboratories for analysis will be performed according to sites guidelines provided by the sponsor.

Complete instructions for processing, labeling, storage and shipping of samples are included in the Serology Guidelines.

3.4 Duration of Subject's Expected Participation in the Entire Study

Each subject will participate in the study for 28 days after vaccination.

3.5 Stopping/Pausing Rules

There are no predetermined stopping rules. However, in case a SUSAR will occur or in case of increased rate of SAE, the sponsor will assess the overall safety profile.

The sponsor or the investigator (following consultation with the sponsor) has the right to discontinue this study at any time. If the clinical study is prematurely terminated, the investigator must promptly inform the study subjects and the EC and should assure appropriate follow-up for the subjects. All procedures and requirements pertaining to the archiving of the documents should be followed. All remaining study materials must be returned to the sponsor.

4.0 SELECTION OF STUDY POPULATION

4.1 Inclusion Criteria

1. Males and females of age ≥18 to ≤40 years.
2. Individuals who, after the nature of the study have been explained to them, have given written consent according to local regulatory requirements.
3. Individuals in good health as determined by the outcome of medical history, physical examination and clinical judgment of the investigator.

4. Individuals with negative screening tests for drug addition as follows:

Opiate	Negative
Cocaine	Negative
Amph/Metamphetamine	Negative
Cannabinoides	Negative

5. If women, use of birth control one month before study start, a negative pregnancy test and willingness to use birth control measures for the entire study duration.

4.2 Exclusion Criteria

1. Individuals with behavioral or cognitive impairment or psychiatric disease that, in the opinion of the investigator, may interfere with the subject's ability to participate in the study.
2. Individuals with any progressive or severe neurological disorder, seizure disorder or Guillain-Barré syndrome.
3. Individuals who are not able to understand and to follow all required study procedures for the whole period of the study.
4. Individuals with history of any illness that, in the opinion of the investigator, might interfere with the results of the study or pose additional risk to the subjects due to participation in the study.
5. Individuals with known or suspected HIV infection or HIV related disease, with history of an autoimmune disorder or any other known or suspected impairment /alteration of the immune system, or under immunosuppressive therapy including use of systemic corticosteroids or chronic use of inhaled high-potency corticosteroids within the previous 30 days, or were in chemotherapy treatment within the past 6 months.
6. Individuals with a known bleeding diathesis, or any condition that may be associated with a prolonged bleeding time.
7. Individuals with any serious chronic or progressive disease according to judgment of the investigator (e.g., neoplasm, insulin dependent diabetes, cardiac, renal or hepatic disease).
8. Individuals who have any malignancy or lymphoproliferative disorder.
9. Individuals with history of allergy to vaccine components.
10. Individuals participating in any clinical trial with another investigational product 30 days prior to first study visit or intent to participate in another clinical study at any time during the conduct of this study.
11. Individuals who have previously received any vaccines against typhoid fever (either oral live attenuated or injectable vaccines)

12. Individuals who received any other vaccines within 4 weeks prior to enrolment in this study or who are planning to receive any vaccine within 4 weeks from the study vaccine
13. Individuals who have received blood, blood products and/or plasma derivatives including parenteral immunoglobulin preparations in the past 12 weeks.
14. Individuals who are part of study personnel or close family members to the personnel conducting this study.
15. Individuals with body temperature $\geq$ 38.0 degrees Celsius within 3 days of intended study immunization.
16. BMI > 35 kg/m^{2}.
17. Individuals with history of substance or alcohol abuse within the past 2 years.
18. Women who are pregnant or breast-feeding or of childbearing age who have not used any birth control measure one month prior to study start or do not plan to use acceptable birth control measures, for the duration of the study.
19. Females with history of stillbirth, neonatal loss, or previous infant with anomaly.
20. Individuals who have a previously ascertained or suspected disease caused by S. Typhi.
21. Individuals who have had household contact with/and or intimate exposure to an individual with laboratory confirmed S. Typhi.
22. Any condition which, in the opinion of the investigator may interfere with the evaluation of the study objectives.

4.3 Withdrawal of Subjects from Therapy or Assessment

The subject can withdraw consent for participation in the study at any time without penalty or loss of benefit to which the subject is otherwise entitled. The investigator can withdraw a subject if, in his or her clinical judgment, it is in the best interest of the subject or if the subject cannot or will not comply with the protocol.

If a subject withdraws from the study, the reason for withdrawal should be documented in the subject's medical record and reported in CRF.

<u>For women of childbearing potential</u>:

Any subject, who, despite the requirement for adequate contraception, becomes pregnant during the trial, will be followed-up. The site should maintain contact with the pregnant subject, complete a "Pregnancy Report" CRF as soon as possible, and obtain pregnancy outcome information for "Pregnancy Follow-up" CRF. In case of withdrawal, the subject should be followed up and the reason for withdrawal (e.g. pregnancy) should be recorded

in detail on the "Study Termination" CRF as well as on the subject's medical records (see section 6.2.5 for further details).

Withdrawn subjects will not be replaced.

5.0 TREATMENT OF SUBJECTS

Subjects will be randomly assigned in a 1:1:1:1 ratio, to receive one of the following vaccines:

1. 0.5 ml dose of 12.5 µg NVGH Vi-CRM_{197} vaccine (Dose A)
2. 0.5 ml dose of 5.0 µg NVGH Vi-CRM_{197} vaccine (Dose B)
3. 0.5 ml dose of 1.25 µg NVGH Vi-CRM_{197} vaccine (Dose C)
4. 0.5 ml dose of licensed Vi polysaccharide vaccine (Typherix®, GlaxoSmithKline)

All study vaccines are to be kept in a secure location with appropriate storage conditions, temperature monitoring, and separate from other vaccines.

5.1 Investigational Vaccine(s)

5.1 Investigational Vaccine - NVGH S. Typhi conjugate vaccine

The test vaccine is NVGH S. Typhi glyconjugate vaccine (Vi-CRM_{197}). The vaccine is available in single dose vials containing 0.7 ml of injectable solution. Each 0.5 ml immunization dose of Vi-CRM_{197} contains 25 µg of Vi polysaccharide conjugated to CRM_{197} (Vi-CRM_{197} - 25 µg) The vaccine does not contain any adjuvant, stabilizer or preservative.

In the present trial, the vaccine will be used at three different antigen concentrations (Vaccine Dose A, Vaccine Dose B and Vaccine Dose C) which will be obtained by bed side mixing of Vi-CRM_{197} - 25 µg.

Vi-CRM_{197} – Dose A

Each 0.5 ml dose of Vi-CRM_{197} – Dose A contains **12.5 µg** of Vi polysaccharide conjugated to CRM_{197}. The vaccine does not contain any adjuvant, stabilizer or preservative.

Vi-CRM_{197} – Dose A is obtained by mixing 0.5 ml of Vi-CRM_{197} - 25 µg with 0.5 ml of saline solution (resulting in 1.0 ml of **12.5 µg** Vi-CRM_{197} – Dose A)

The subjects randomized to receive Vi-CRM_{197} – Dose A will receive one 0.5 ml IM injection in the deltoid of the non-dominant arm (Group A).

Vi-CRM$_{197}$ – Dose B

Each 0.5 ml dose of Vi-CRM$_{197}$ – Dose B contains **5.0 µg** of Vi polysaccharide conjugated to CRM$_{197}$. The vaccine does not contain any adjuvant, stabilizer or preservative.

Vi-CRM$_{197}$ – Dose B is obtained by mixing 0.5 ml of Vi-CRM$_{197}$ - 25 µg with 2.0 ml of saline solution (resulting in 2.5 ml of **5 µg** Vi-CRM$_{197}$ – Dose B)

The subjects randomized to receive Vi-CRM$_{197}$ – Dose B will receive one 0.5 ml IM injection in the deltoid of the non-dominant arm (Group B).

Vi-CRM$_{197}$ – Dose C

Each 0.5 ml dose of Vi-CRM$_{197}$ – Dose C contains **1.25 µg** of Vi polysaccharide conjugated to CRM$_{197}$. The vaccine does not contain any adjuvant, stabilizer or preservative.

Vi-CRM$_{197}$ – Dose C is obtained by mixing 0.5 ml of Vi-CRM$_{197}$ - 25 µg with 9.5 ml of saline solution (resulting in 10.0 ml of **1.25 µg** Vi-CRM$_{197}$ – Dose C).

The subjects randomized to receive Vi-CRM$_{197}$ – Dose C will receive one 0.5 ml IM injection in the deltoid of the non-dominant arm (Group C).

For additional information please refer to the investigator brochure provided.

5.2 Control Vaccine – GSK Typherix®

The licensed Vi polysaccharide vaccine (Typherix®, GlaxoSmithKline) is a clear isotonic colourless solution.

Vi antigen is extracted from the bacterial capsule of S. Typhi strain TY2. A 0.5 ml dose of Vi polysaccharide vaccine contains 25 µg of Vi. One 0.5 ml dose out of single dose pre-filled syringes of Vi polysaccharide vaccine will be injected IM in the deltoid of the non-dominant arm.

For additional information please refer to the vaccine package insert provided.

5.3 Concomitant Vaccines or Treatment

No concomitant vaccines will be studied as part of this trial.

No concomitant vaccines are allowed during the 4 weeks period before and after vaccination (Visit 1). Subjects who received any other vaccines within 4 weeks prior to study vaccination or who are planning to receive any vaccine within 4 weeks after are excluded from the study (see exclusion criteria #12).

5.4 Vaccines Preparation and Administration

Three doses of NVGH Vi-CRM$_{197}$ will be used in this study:

- **Vi-CRM$_{197}$ – Dose A** is obtained by mixing 0.5 ml of Vi-CRM$_{197}$ - 25 µg with 0.5 ml of saline solution (resulting in 1.0 ml of **12.5 µg** Vi-CRM$_{197}$ – Dose A)
- **Vi-CRM$_{197}$ – Dose B** is obtained by mixing 0.5 ml of Vi-CRM$_{197}$ - 25 µg with 2.0 ml of saline solution (resulting in 2.5 ml of **5.0 µg** Vi-CRM$_{197}$ – Dose B)
- **Vi-CRM$_{197}$ – Dose C** is obtained by mixing 0.5 ml of Vi-CRM$_{197}$ - 25 µg with 9.5 ml of saline solution (resulting in 10.0 ml of **1.25 µg** Vi-CRM$_{197}$ – Dose C)

The bedside mixing procedure to obtain Vi-CRM$_{197}$ – Dose A, B and C will be performed by a trained unblinded site staff. The administration of the vaccines to subjects enrolled into the study will only be done by the unblinded site staff according to the procedures stipulated in this study protocol. The unblinded site staff responsible for vaccine administration will be composed only by personnel who are qualified to perform that function under applicable local laws and regulations for the specific study site.

The licensed vaccine must be prepared according to the package insert before use.

For further details please refer to the H01_04TP Study Manual.

PRECAUTIONS TO BE OBSERVED IN ADMINISTERING STUDY VACCINE:

The vaccination site should be disinfected with a skin disinfectant (e.g., 70% alcohol). Before vaccination, the skin must be dry. **DO NOT inject intravascularly**.

Standard immunization practices should be observed and care should be taken to administer the injection intramuscularly. As with all injectable vaccines, appropriate medical treatment and supervision should be readily available (medication available in the room where the vaccines are administered), in case of anaphylactic reactions within the 1 hour observation period following administration of the study vaccine. According to the Belgian guidelines (2004) from the Higher Health Council, epinephrine 1:1000 should be available in case of any anaphylactic reactions.

Study vaccines should not be administered to individuals with known hypersensitivity to any component of the vaccines.

Vaccinations must not be administered to any subject with a clinically significant active infection (as assessed by the investigator) or measured by body (axillary) temperature ≥ 38.0°C/100.4ºF within 3 days of the intended date of vaccination. If either of these is observed, vaccination should be postponed until the subject's temperature remains below 38.0°C/100.4°F for at least 3 days or the investigator feels that the subject's illness has stabilized, as appropriate.

5.5 Other Concomitant Treatment or Vaccines

The following medications will be recorded in CRF:

- Prescription and non prescription medications taken by the subject for the treatment of any SAEs or AEs after vaccination (Visit 1) till end of study (Visit 2).
- All vaccinations received by the subject after vaccination (Visit 1) till end of study (Visit 2).
- Immunosuppressants or other immune-modifying drugs, investigational drugs/vaccines, immunoglobulins or any blood products, as well as any medications taken by subject as treatment for SAEs after vaccination (Visit 1) till end of study (Visit 2).

Although protocol violations to be treated as such, also the following medications will have to be recorded in the CRF if they occur:

- Investigational products taken by the subject after vaccination (Visit 1) till end of study (Visit 2).
- Vaccines received by the subject after vaccination (Visit 1) till end of study (Visit 2).
- Blood, blood products and/or plasma derivates including parenteral immunoglobulin preparations taken by the subject after vaccination (Visit 1) till end of study (Visit 2).
- Prior medications include (at a minimum) all prescription medications taken regularly by a subject at the time of study enrolment.

Use of the following concomitant medications after enrolment may interfere with the interpretation of the study objectives or indicate an underlying condition resulting in a major protocol violation according to the medical judgment of the investigator and NVGH physician **(Dr. Audino Podda, phone:+39 0577 243496)**:

- Systemic Corticosteroids
- Blood or blood products

5.6 Vaccination Compliance

The investigator is responsible for adequate and accurate accounting of vaccine usage. The investigator or designee will administer the study vaccines only to individuals included in this study following the procedures set out in this study protocol. The date, dosage, and time of the vaccinations must be recorded. The investigator must track vaccines received, used and wasted and will retain all unused or expired products as described in section 3.3.4.

6.0 EFFICACY/IMMUNOGENICITY AND SAFETY ASSESSMENTS

6.1 Appropriateness of Measurements

For immunogenicity: The measures of immunogenicity used in this study are standard, i.e., widely used and generally recognized as reliable, accurate, and relevant (able to describe the quality and extent of the immune response).

For safety: The measures of safety used in this study are a close vigilance for, and stringent reporting of, selected local and systemic reactions routinely monitored in vaccine clinical trials as indicators of reactogenicity, and of adverse events and serious adverse events in the entire duration of the study.

6.1.1 Immunogenicity

The measure of immune response is the anti-Vi ELISA antibody concentration obtained 28 days after vaccination (Visit 2, Day 28).

The serologic assays on clinical samples will be performed at Novartis Vaccines, Clinical Serology Laboratory, Marburg, Germany or a delegated laboratory.

6.1.2 Methods, Criteria and Timing for Assessing and Recording Immunogenicity Parameters

Blood samples for immunogenicity testing are obtained at Visit 1 (Day 1) and Visit 2 (Day 28). The measure of immune response is the anti-Vi ELISA antibody concentration obtained at Day 28 (28 days after the injection).

6.2 Safety Parameters

On Visit 2, the Investigator should question the subject about any medication taken. Investigator will record medications (as instructed in Table 6.2.1) in the CRF with trade name and/or generic name, medical indication, total daily dose, route of administration, start and end dates of treatment.

Table 6.2.1: Medical and Safety Assessments to be reported into CRF

**Before Visit 1 vaccination**	**Medical History:**<br>Any significant past diagnoses in the last 12 months including injuries, hospitalizations, major surgeries, or other significant medical conditions which may impair the assessment of immunogenicity or safety of the study vaccine
	**Medications:**<br>All vaccinations received in the 4 weeks before study vaccination.<br>All investigational products received during the previous 30 days

	Blood , blood products and /or plasma derivates including parenteral immunoglobulin preparations during the previous 12 weeks
**During 1 hour after vaccination**	**Immediate reaction:**<br>Signs or symptoms of anaphylaxis
	**Local reactions:**<br>Pain, Erythema, Induration
	**Systemic reactions:**<br>Chills, Malaise, Myalgia, Headache, Arthralgia, Fatigue
**During 7 days after vaccination (Day 1 to Day 7)**	**Temperature:**<br>Axillary temperature (Fever is defined as axillary temperature ≥ 38.0 °C),
	**Local reactions:**<br>Pain, Erythema, Induration
	**Systemic reactions:**<br>Chills, Malaise, Myalgia, Headache, Arthralgia, Fatigue
**All study (for 28 days)**	**All Adverse and Serious Adverse Events:**<br>All AEs or/and SAEs must be documented and followed up until the event is resolved, subsided, stabilized, disappeared or is otherwise explained or the subject is lost to follow-up. *If AE or/and SAE remains unresolved at study termination, a clinical assessment will be made by the investigator and the sponsor Medical Monitor to determine whether continued follow up of the* AEs or/and SAEs *is warranted.*<br><br>Any SAE occurring at any time outside the 28 days study period and considered to be caused by the study vaccine will be reported.
	**Medications:**<br>Any medications administered as treatment of AEs or/and SAEs, immunosuppressants or other immune-modifying drugs, investigational drugs/vaccines, immunoglobulins or any blood products.

A brief medical history will be obtained and physical examination performed for each subject entered into the study.

Solicited reactogenicity, including local and systemic reactions and other unsolicited adverse events will be collected in the study, as detailed in sections 6.2.1 to 6.2.5 and Table 6.2.1.

Reactogenicity will be assessed daily during the first week following vaccination: local and systemic reactions occurring at day 1 to day 7 will be recorded in the diary card. The overall safety follow-up will be 28 days for each subject: all adverse events (AE) and serious adverse events (SAE) will be collected and documented for the duration of the study (28 days).

6.2.1 Local and Systemic Reactions

The occurrence of selected indicators of reactogenicity (listed in Table 6.2.1) and other unsolicited adverse events will be recorded in the diary card by the subject and reported in the corresponding CRF.

6.2.2 Adverse Events

An adverse event (AE) is defined as any untoward medical occurrence in a subject or clinical investigation subject administered a pharmaceutical product at any dose that does not necessarily have to have a causal relationship with this treatment. An AE can, therefore be any unfavorable and unintended sign (including an abnormal laboratory finding, for example), symptom, or disease temporally associated with the use of an investigational product, whether or not considered related to the investigational product. This definition includes intercurrent illnesses or injuries and exacerbation of pre-existing conditions.

An unexpected adverse event is one that is not listed in the current Summary of Product Characteristics or the Investigator's Brochure or an event that is by nature more specific or more severe than a listed event.

All AEs will be monitored until resolution or, if the AE becomes chronic, a cause identified. If an AE is unresolved at the conclusion of the study, a clinical assessment will be made by the investigator and medical monitor whether continued follow-up of the AE is warranted.

The severity of events reported on the "Adverse Events" CRF will be determined by the investigator as:

Mild: transient with no limitation in normal daily activity.
Moderate: some limitation in normal daily activity.
Severe: unable to perform normal daily activity.

The relationship of the study treatment to an AE will be determined by the investigator based on the following definitions:

1. Not Related

The AE is not related to an investigational vaccine if there is evidence that clearly indicates an alternative explanation. If the subject has not received the vaccine, the timing of the exposure to the vaccine and the onset of the AE are not reasonably related in time, or other facts, evidence or arguments exist that strongly suggest an alternative explanation, then the AE is not related.

2. Possibly Related

The administration of the investigational vaccine and AE are considered reasonably related in time **and** the AE could be explained either by exposure to the investigational vaccine or by other causes.

3. Probably Related

Exposure to the investigational vaccine and AE are reasonably related in time and no alternative explanation has been identified.

The relationship of the study treatment to an AE will be determined by the investigator.

6.2.3 Serious Adverse Events

A serious adverse event (SAE) is defined as any untoward medical occurrence that at any dose:

- Results in death.
- Is life-threatening (i.e., the subject was, in the opinion of the investigator, at immediate risk of death from the event as it occurred); it does not refer to an event which hypothetically might have caused death if it were more severe.
- Requires or prolongs subject’s hospitalization.
- Results in persistent or significant disability/incapacity (i.e., the event causes a substantial disruption of a person’s ability to conduct normal life functions).
- Results in a congenital anomaly/birth defect.
- Requires intervention to prevent permanent impairment or damage.
- Is an important and significant medical event that may not be immediately life threatening or resulting in death or hospitalization but, based upon appropriate medical judgment, may jeopardize the subject or may require intervention to prevent one of the other outcomes listed above.

Grade 4 and 5 laboratory deviations are also considered SAEs.

Of note: a “possible vaccine failure” should be reported as a SAE only if it resulted in a laboratory confirmed infectious disease which should have been prevented by the vaccine implied.

Adverse events which do not fall into these categories are defined as **non-serious**.

It should be noted that a severe adverse event need not be serious in nature and that a serious adverse event need not, by definition, be severe.

In addition, a pre-existing event or condition that results in hospitalization should be recorded on the Medical History CRF. If the onset of an event occurred before the subject entered the trial (e.g., any pre-planned hospitalization for conditions like cosmetic treatments or for non-emergency routine visits for a pre-existing condition), the hospitalization would not lead to an AE being classified as serious unless, in the view of the investigator, hospitalization was prolonged as a result of participation in the clinical trial or was necessary due to a worsening of the pre-existing condition.

For instructions on SAE reporting, please refer to section 6.2.6.

6.2.4 Subject's Diary Card

Diary Cards will be completed by the subject and used by the study staff to record safety information (concomitant medications and vaccinations, local and systemic reaction, AE and SAE) occurring in the entire duration of the study (28 days).

According to ICH GCP guidelines 1.52, subject's Diary Card is a "Source Document". It is an investigator responsibility to ensure that data on the diary card correspond to the real health status of subject and to accurately transcribe data from the Diary Card to the CRF.

In case the Diary Card is not available, the study staff will document the reasons for the missing documents on the Comment section of the CRF and the CRF will be completed with UNK for all assessments. Nevertheless, if a SAE occurred it must be recorded in SAE form and in the adverse event section of CRF.

6.2.5 Pregnancies

To ensure subjects' safety, each pregnancy in a subject on study vaccine must be reported to Novartis Vaccines Institute for Global Health within 24 hours of learning of its occurrence. The pregnancy should be actively followed up to determine outcome, including spontaneous or voluntary termination, details of the birth, and the presence or absence of any birth defects, congenital abnormalities, or maternal and/or newborn complications.

Any SAE experienced during pregnancy must be reported on the SAE Report Form.

6.2.6 Methods and Timing for Assessing and Recording Safety Parameters

The period of observation for adverse events starts from the time the subject receives the vaccination. All study subjects will be observed at site for at least 1 hour after vaccination for evidence of immediate reactions and in particular for symptoms of allergic phenomena (such as rashes or other allergic manifestations). Each subject will be instructed to complete a diary card for 28 days (duration of study) following vaccination, to describe local and systemic reactions (during the 7-day period after vaccination) and AEs/ SAEs (during the 28 days duration of the study).

On day 8, all information on local or systemic reactions and adverse events (AEs) recorded in the diary cards will be collected on an ad-hoc form via a structured telephone call. The ad-hoc form will be kept as a source documentation for reconciliation with information in diary card brought in by the subject at visit 2 (day 28).

All adverse events occurring within the above period, regardless of severity, will be monitored by the investigator until resolution. All subjects experiencing adverse events - whether considered associated with the use of the study vaccine or not - must be monitored until symptoms subside and any abnormal laboratory values have returned to baseline, or until there is a satisfactory explanation for the changes observed, or until death, in which case a full pathologist's report should be supplied, if possible. All findings must be reported on an "Adverse Events" CRF and on the "Vaccine Serious Adverse Event" form, if necessary. All findings in subjects experiencing adverse events must be reported also in the subject's medical records.

All SAEs which occur during the course of the trial, whether considered to be associated with the study vaccination or not, must be reported **within 24 hours** or at the latest on the following working day by telephone or fax to Novartis Vaccines Institute for Global Health. The "Vaccine Serious Adverse Event" form is to be completed for all SAEs and faxed to the sponsor.

Study Contacts for Reporting Serious Adverse Events

Audino Podda, MD
Novartis Vaccines Institute for Global Health (NVGH)
Via Fiorentina, 1 - 53100 Siena, Italy
Phone:+39 0577 243496
Fax: +39 0577 539114
Mobile: +39 335 7026950
Email: audino.podda@novartis.com

The original form is retained by the investigator. The event is also documented on the "Adverse Events" CRF. After receipt of the initial report, the Physician/ Site CRA will review the information and contact the investigator if it is necessary to obtain further information for assessment of the event. Any medication or other therapeutic measures used to treat the event will be recorded on the appropriate CRF(s) in addition to the outcome of the AE. Any serious adverse reaction must be reported to the EC or IRB by the Site in a timely manner. Adequate documentation will be provided to the sponsor showing that the EC or IRB has been properly notified. The sponsor must also comply with the applicable regulatory requirement(s) related to the reporting of unexpected serious and non-serious adverse vaccine reactions to the regulatory authority(ies) and the IRB/IEC.

If required, a follow-up report including all new information obtained on the serious adverse event must be prepared and sent to NVGH. The report should be marked "Follow-up report."

The investigator will submit, on request, copies of all these reports to the EC or IRB and other relevant authorities.

Post-Study Events

Any SAE occurring at any time after the end of the study and considered to be caused by the study vaccine - and therefore a possible adverse reaction - must be reported by using a SAE Report Form to:

Audino Podda, MD
Novartis Vaccines Institute for Global Health (NVGH)
Via Fiorentina, 1 - 53100 Siena, Italy
Phone:+39 0577 243496
Fax: +39 0577 539114
Mobile: +39 335 7026950
Email: audino.podda@novartis.com.

6.3 Data Monitoring Committee

A Data Safety Monitoring Board (DSMB) will not be convened for this study.

7.0 STATISTICAL PLAN

7.1 Statistical Hypothesis

This Phase 2 safety and immunogenicity trial is aimed to descriptively evaluate the safety and immunogenicity profiles of the study vaccines. No specific hypotheses are tested in this trial.

7.2 Sample Size and Power Considerations

Given the sample size selected for this trial, the precision to define certain incidence rates and the power to detect differences in the incidence rates between the vaccine groups in ad hoc or exploratory analyses will be indicated in the following examples:

- For each vaccine, with 20 subjects per vaccine group, there is a 90% probability of observing at least 1 subject with an adverse event if the true rate of such an event is 10.9%.

The probability to detect at least one subject with an adverse event (for each of the vaccine groups) is reported in Table 1 by underlying rates of AEs descending from 15% - 1.5%:

Table 1

Underlying Event Rate (Frequency of AE)	Probability of Detection N=20
15%	96%
12.5%	93%
10%	88%
7.5%	79%
5%	64%
2.5%	40%
1.5%	26%

- Comparative statistics for the safety variables will not be calculated. The study has low power to detect anything other than large differences in the incidence of local and systemic reactions between vaccination groups. In fact, a difference between a 10% reaction rate (n=20) in one of the vaccine groups and a 20% reaction rate in one of the other vaccine groups (n=20) is detectable with only 7% power at a 5% two-sided alpha level. Safety comparisons, with an 80% power and a 5% two-sided alpha level, will permit detection of the differences (calculated by using the Fisher's exact test) reported in Table 2:

Table 2

Underlying Event Rate in One Vaccine Group	Difference Detected with a 80% Power
30%	48.0%
20%	47.5%
10%	43.0%
5%	39.5%
2.5%	36.0%

- The precision of the estimates, expressed by the 95% Clopper-Pearson CI, for several possible observed values of the percentages of seroconversion for each individual vaccine group is shown in Table 3:

Table 3

**Observed Number of Responders (Response Rate) n (% of N=20)**	**95% CI (N = 20) % %**
10 (50%)	27.2 - 72.8
12 (60%)	36.1 - 80.9
14 (70%)	45.7 – 88.1
16 (80%)	56.3 – 94.3
18 (90%)	68.3 – 98.8
19 (95%)	75.1 - 99.9
20 (100%)	83.2 - 100.0

- A sample size of 20 subjects per group will permit the detection of a 2.5-fold difference in geometric mean antibody concentration between two vaccine groups with at least 86% power at two-sided alpha=0.05 when the standard deviation of the log_{10} concentrations is no greater than 0.4. This same sample size of 20 subjects per group will permit the detection of a 2.5-fold difference in geometric mean antibody concentration between the three combined dose groups and the control group (N=60 versus N=20) with at least 80% power at two-sided alpha = 0.05 if the standard deviation of the log_{10} concentrations is no greater than 0.54.

7.3 Population for Analysis

Definition of populations to be analyzed:

a) *All Randomized Population*

- all subjects who:
  - signed informed consent
  - are randomized into the study.

  This population will be used for the analysis of demographics and all subject listings.

b) Immunogenicity Population -- Modified Intention-to-Treat (mITT) population

- all subjects in the randomized population who:

- actually receive study vaccination, and
- provide an evaluable serum sample at Visit 2.

c) Immunogenicity Population -- Per Protocol (PP) population

- all subjects in the mITT Immunogenicity population who:
  - receive the correct vaccine as assigned in the randomization list
  - provide an evaluable serum sample at Visit 2, and
  - have no major protocol violation as defined prior to unblinding.

A major deviation is defined as a protocol deviation that is considered to have significant impact on the immunogenicity result of the subject.

d) Safety population

- all randomized subjects who:
  - receive study vaccine
  - provide post-vaccination safety data

7.4 Analysis of Demographic and Baseline Characteristics

Descriptive statistics (mean, standard deviation, median, minimum and maximum) for age, height and weight at enrolment will be calculated overall and by vaccine group.

Distributions of subjects by sex and ethnic origin will be summarized overall and by vaccine group.

7.5 Analysis of Efficacy/Immunogenicity Endpoints

7.5.1 Description of Response Variables

The primary response variable is the Vi specific antibody level measured 28 days after the vaccination as assessed by ELISA.

7.5.2 Statistical Methods for Efficacy/Immunogenicity Variables

The measures of immunogenicity, against the Vi antigen of S.Typhi, will include:

- Geometric mean concentrations (GMCs), pre- and post-vaccination, as determined by ELISA, and applicable geometric mean ratios between post- and pre-vaccination samples. GMCs and associated 95% confidence intervals (CIs) will be computed by exponentiating (base 10) the means of the log-transformed (base 10) titers and their 95% confidence intervals.

- Seroconversion rate: percentage of subjects achieving at least a four-fold rise in ELISA antibody concentration in the post-vaccination blood sample. The associated 95% CI will be calculated using the Clopper-Pearson method.

Summaries of GMCs and the seroconversion rate will be presented by vaccine group.

The assessment of these endpoints will be descriptive. Therefore, no specific criteria for evaluation have been set.

7.6 Analysis of Safety (Endpoints) and Tolerability

The measures of safety will include:

- Deviations from normal values (see Appendix II) of hematological and haematochemical blood tests and urinalysis after immunization. Clinical significance of abnormal values may be assessed by medical judgment.

- Numbers and percentage of subjects with solicited local and systemic adverse reactions as well as numbers and percentage of subjects with reported unsolicited adverse events and serious adverse events.

Solicited local reactions include erythema, induration and pain at injection site; solicited systemic reactions include headache, arthralgia, chills, fatigue, malaise, myalgia, and fever as measured by axillary temperature for Day 1 through 7 of the study.

All local and systemic reactions will be collected for 7 days after immunization (i.e., days 1 to 7). All AEs and SAEs will be collected for 28 days.

All SAEs and AEs (including onset of chronic illness) will be judged by the Investigator as either probably related, possibly related, or not related to vaccine and will be tabulated. All SAEs and AEs resulting in withdrawal from the study will be summarized.

7.6.1 Analysis of Extent of Exposure

Not applicable.

7.6.2 Analysis of Local and Systemic Reactions

Frequencies and percentages of subjects experiencing each reaction will be presented for each symptom severity. Summary tables showing the occurrence of any local or systemic reaction overall and at each time point will also be presented.

Post-vaccination reactions reported from day 1 to day 7 will be summarized by maximal severity and by vaccine group. The severity of local reactions, including injection-site

erythema and induration will be categorized as none, 1 to≤10 mm, 11 to ≤25 mm, 26 to ≤50mm, 51 to ≤100 mm and >100 mm (severe local reactions).

The severity of pain and systemic reactions (e.g., chills, malaise, myalgia, headache, etc. as per section 6.2.1) occurring up to 7 days after vaccination will be categorized as none, mild (transient with no limitation in normal daily activity), moderate (some limitation in normal daily activity), and severe (unable to perform normal daily activity).

Axillary temperature will be categorized as <38°C (no fever), 38-38.9°C (mild), 39-39.9°C (moderate) and ≥40°C (severe).

No statistical inference will be performed for the local and systemic reaction safety variables.

7.6.3 Analysis of Other Adverse Events

All the adverse events occurring during the study, judged either as related to vaccination or not by the investigator, will be recorded as specified in section 6.2.5. The original verbatim terms used by investigators to identify adverse events in the CRFs will be mapped to preferred terms using the MedDRA dictionary. The adverse events will then be grouped by MedDRA preferred terms into frequency tables according to system organ class. All reported adverse events, as well as adverse events judged by the investigator as at least possibly related to study vaccine, will be summarized according to system organ class and preferred term within system organ class. These summaries will be presented by vaccination group. When an adverse event occurs more than once for a subject, the maximal severity and strongest relationship to the vaccine group will be counted. Additionally, three separate summaries will be produced: (i) serious adverse events, (ii) adverse events that are possibly or probably related to vaccine, and (iii) adverse events that are unrelated to vaccine. Data listings of all adverse events will be provided by subject. In addition, a listing of subjects withdrawn from the study because of an adverse event will be presented.

7.7 Planned Interim Analysis

No interim analysis will be performed.

8.0 STUDY MONITORING, AUDITING AND DOCUMENTATION

Study monitoring and auditing will be performed in accordance with the sponsor's procedures and applicable regulatory requirements (e.g. EMEA, ICH and GCP guidelines).

Investigators and/or their study staff will be trained on the study protocol and all applicable study procedures prior to subject enrolment. CRFs supplied by the sponsor must be completed for each enrolled subject. The data entries as well as study related documents will be checked by the sponsor and/or trained delegates of the sponsor.

8.1 Study Monitoring

Study progress will be monitored by Novartis Vaccines Institute for Global Health or its representative (e.g. a contract research organization) as frequently as necessary to ensure the rights and well-being of study subjects are protected; to verify adequate, accurate and complete data collection; protocol compliance and to determine that the study is being conducted in conformance with applicable regulatory requirements. Arrangements for monitoring visits will be made in advance in accordance with the monitoring plan, except in case of emergency.

8.2 Source Data Verification

Data recorded on the CRF will be verified by checking the CRF entries against source documents (i.e., all original records, laboratory reports, medical records, subjects diaries, memory aids) in order to ensure data completeness and accuracy as required by study protocol. The investigator and/or site staff must make CRFs and source documents of subjects enrolled in this study available for inspection by NVGH or its representative at the time of each monitoring visit.

At a minimum, source documentation must be available to substantiate subject identification, eligibility and participation, proper informed consent procedures, dates of visits, adherence to protocol procedures, adequate reporting and follow-up of adverse events, administration of concomitant medication, study vaccine receipt/dispensing/return records, study vaccine administration information, and date of completion and reason. Specific items required as source documents will be reviewed with the investigator before the study.

The source documents must also be available for inspection, verification and copying, as required by regulations, by officials of the regulatory health authorities (e.g. FDA, EMEA and others) and/or ECs/IRBs. The investigator and study site staff must comply with applicable privacy, data protection and medical confidentiality laws for use and disclosure of information related to the study and enrolled subjects.

The subject must also allow access to her medical records. Each subject should be informed of this prior to the start of the study.

9.0 DATA MANAGEMENT

Electronic CRF

An electronic data capture (EDC) system (e.g., Inform™) will be used to expedite the entry of data. The investigator will enter data into the web enable EDC system in a timely manner; the data will be stored in clinical database management system. eCRF data will be reviewed routinely by data management and clinical monitors. In case the e CRF is not available, collection of clinical data will be done in a paper CRF.

Electronic Data Transfer (EDT) is one method being used by NVGH for collecting laboratory data. The laboratory (e.g., central laboratory) will send data as electronic files by a secured method (e.g., via diskette, CD, as an encrypted file attachment on electronic mail, or as a direct transfer into a specified server directory) to data management department. The data file is pre-processed and loaded by the data management Lab Manager into the study database. The laboratory will submit a results' file containing the tests and the results as specified in the protocol. If the laboratory provides the service, it will also submit a Demography (DEMOG) file containing the subject's demographic information. If the file includes results of data blinded to Clinical, the source will provide a separate RESULTS file that will be loaded into a separate laboratory table.

All serology data analyzed by serology laboratory will be entered into the Seroad database by the Serology Laboratory. All results will be checked in the laboratory for validity and completeness.

For this protocol, antibody laboratory data and/or safety laboratory data will be transmitted via EDT.

9.1 Data Handling Procedures

Coding will be performed using the following dictionaries:

Adverse Events:	MedDRA
Concomitant illness:	ICD-9
Concomitant and intercurrent therapy:	WHO Drug Dictionary

9.2 Documentation of Study Findings

The investigator must review and electronically sign the eCRFs to verify their accuracy. Correction to data on eCRFs will be tracked via an audit trail within InForm™, web based electronic data capture system. Each correction will be identified by the person making the change and will include time, date, and reason for change. If corrections are

made to a previously and electronically signed CRF, the investigator must confirm and endorse the changes.

As part of the conduct of the trial, Novartis Vaccines Institute for Global Health may have questions about the Case Report Form data after the site has entered the data. These questions will be raised within InForm™. The Investigator will provide follow-up clarification and/or resolution of data issues raised by the monitor or the data manager.

An explanation must be provided and documented by the investigator for all missing data.

In case the e CRF is not available, collection of clinical data will be done in a paper CRF.

9.3 Data Protection

Novartis Vaccines Institute for Global Health respects the subjects' rights to privacy and will ensure the confidentiality of their medical information in accordance with all applicable laws and regulations.

The sponsor as Data Controller according to the European Directive on the protection of individuals with regard to the processing of personal data and on the free movement of such data [95/46/EC] confirms herewith compliance to Directive 95/46/EC in all stages of Data Management.

10.0 RECORD RETENTION

Investigators must retain all study records required by NVGH and by the applicable regulations in a secure and safe facility. The investigator must consult a NVGH representative before disposal of any study records, and must notify the sponsor of any change in the location, disposition, or custody of the study files. Essential documents must be retained until at least 2 years after the last approval of a marketing application in an ICH region and until there are no pending or contemplated marketing applications in an ICH region or at least 2 years have elapsed since the formal discontinuation of clinical development of the investigational product. "Essential documents" are defined as documents that individually and collectively permit evaluation of the conduct of a trial and the quality of the data produced. These documents should be retained for a longer period however, if required by the applicable regulatory requirements or by an agreement with the sponsor or The Committee for Medicinal Products for Human Use (CHMP) requires retention for the maximum period of time permitted by the institution, but not less than 15 years (ICH E6, 4.9.5). It is the responsibility of the sponsor to inform the investigator/institution as to when these documents no longer need to be retained (ICH E6, 5.5.12).

11.0 USE OF INFORMATION AND PUBLICATION

Novartis Vaccines Institute for Global Health assures that the key design elements of this protocol will be posted in a publicly accessible database such as clinicaltrials.gov.

12.0 ETHICS

12.1 Regulatory and Ethical Compliance

This clinical study was designed and shall be implemented and reported in accordance with the ICH Harmonized Tripartite Guidelines for Good Clinical Practice, with applicable local regulations (including European Directive 2001/20/EC, US Code of Federal Regulations Title 21, and Japanese Ministry of Health, Labor, and Welfare), and with the ethical principles laid down in the Declaration of Helsinki.

12.2 Informed Consent Procedures

Eligible subjects may only be included in the study after providing written (witnessed, where required by law or regulation), IRB/IEC/REB-approved informed consent, or, if incapable of doing so, after such consent has been provided by a legally acceptable representative of the subject. Informed consent must be obtained before conducting any study-specific procedures (i.e. all of the procedures described in the protocol). The process of obtaining informed consent should be documented in the subject source documents.

Novartis Vaccines Institute for Global Health will provide to investigators a separate document with a proposed informed consent form that complies with the ICH GCP guideline and regulatory requirements and is considered appropriate for this study. Any changes to the proposed consent form suggested by the investigator must be agreed to by NVGH before submission to the IRB/IEC/REB, and a copy of the approved version must be provided to the NVGH monitor after IRB/IEC/REB approval.

Women of child bearing potential should be informed that taking the study vaccine may involve unknown risks to the fetus if pregnancy were to occur during the study and agree that in order to participate in the study they must adhere to the contraception requirements indicated in the protocol for the duration of the study. If case of doubts on the ability of a subject to adhere to these requirements, that subject should not be enrolled in the study.

12.3 Responsibilities of the Investigator and IRB/IEC/REB

The protocol and the proposed informed consent form must be reviewed and approved by a properly constituted Institutional Review Board/Independent Ethics Committee/Research Ethics Board (IRB/IEC/REB) before study start. A signed and dated statement that the protocol and informed consent have been approved by the IRB/IEC/REB must be given to NVGH before study initiation. Prior to study start, the

investigator is required to sign a protocol signature page confirming his/her agreement to conduct the study in accordance with the protocol and all of the instructions and procedures referred to in this protocol and to give access to all relevant data and records to NVGH monitors, auditors, designated agents of NVGH, IRBs/IECs/REBs, and regulatory authorities as required. If an inspection of the clinical site is requested by a regulatory authority, the investigator must inform NVGH immediately that this request has been made.

12.4 Protocol Adherence

Investigators will apply due diligence to avoid protocol deviations. Under no circumstances should the investigator contact NVGH or its agents, if any, monitoring the trial to request approval of a protocol deviation, as no authorized deviations are permitted. If the investigator feels a change to the protocol would improve the conduct of the study this must be considered a protocol amendment, and unless such an amendment is agreed upon by NVGH and approved by the IRB/IEC/REB it cannot be implemented. All significant protocol deviations will be recorded and reported in the CSR.

12.5 Protocol Amendments

An amendment is a written description of change(s) to or formal clarification of a study protocol which may impact the conduct of the clinical study, potential benefit of the clinical study, or may affect subject safety, including changes of study objectives, study design, subject population, sample sizes, study procedures, or significant administrative aspects. An administrative change of a study protocol is a minor correction or clarification that has no significant impact on the way the clinical study is to be conducted and no effect on subject safety (e.g., change of telephone number(s), logistical changes). Protocol amendments must be approved by NVGH, Health Authorities where required, and the IRB/IEC/REB. In cases when the amendment is required in order to protect subjects' safety, the amendment can be implemented prior to IRB/IEC/REB approval. Notwithstanding the need for formal approval of a protocol amendment, the investigator is expected to take any immediate action required for the safety of any subject included in this study, even if this action represents a deviation from the protocol. In such cases, NVGH should be notified of this action and the IRB/IEC/REB at the study site should be informed within 10 working days.

13.0 REFERENCE LIST

(1) Typhoid vaccines: WHO position paper. Weekly epidemiological record 6[83], 49-60. 8-2-2008.

(2) Keitel WA, Bond NL, Zahradnik JM, Cramton TA, Robbins JB. Clinical and serological responses following primary and booster immunization with Salmonella typhi Vi capsular polysaccharide vaccines. Vaccine 1994; 12(3):195-199.

(3) Dintzis RZ. Rational design of conjugate vaccines. Pediatr Res 1992; 32(4):376-385.

(4) Lin FY, Ho VA, Khiem HB, Trach DD, Bay PV, Thanh TC et al. The efficacy of a Salmonella typhi Vi conjugate vaccine in two-to-five-year-old children. N Engl J Med 2001; 344(17):1263-1269.

(5) Kanungo S, Dutta S, Sur D. Epidemiology of typhoid and paratyphoid fever in India. J Infect Dev Ctries 2008; 2(6):454-460.

(6) Zhang XL, Jeza VT, Pan Q. Salmonella typhi: from a human pathogen to a vaccine vector. Cell Mol Immunol 2008; 5(2):91-97.
